# Association between anthropometric criteria and body composition among children aged 6–59 months with uncomplicated severe acute malnutrition: A prospective cohort study from South Sudan

**DOI:** 10.1371/journal.pone.0356530

**Published:** 2026-09-15

**Authors:** Gabriele Rossi, Rachel M. Creighton, Patricia Ayebazibwe, Santo Garang, Mohammed Kamruzzaman, Taesung Park, Namseon Beck, Wendy Dyment

**Affiliations:** 1 Medair Global Support Office, Ecublens, Switzerland; 2 Medair South Sudan Country Program, Juba, South Sudan; 3 Department of Statistics, Seoul National University, Seoul, Korea; Christian Medical College, INDIA

## Abstract

**Background:**

Severe acute malnutrition (SAM) is diagnosed based on bilateral edema, mid‑upper arm circumference (MUAC), and/or weight‑for‑height z‑score (WHZ). These criteria do not fully overlap and may identify children with distinct biological and clinical profiles. Understanding how children with SAM identified by different criteria differ in body composition during outpatient therapeutic program (OTP) treatment may help inform treatment and post‑discharge practices.

**Methods:**

We conducted a prospective cohort study of children aged 6–59 months with uncomplicated SAM admitted by MUAC only (MUAC group), WHZ only (WHZ group), or combined MUAC and WHZ criteria (Combined group) in Aweil Centre, South Sudan. Anthropometry, triceps skinfold thickness, and bioelectrical impedance analysis were collected at admission (n = 194) and discharge (n = 127). Fat mass (FM), fat‑free mass (FFM), fat mass index (FMI), fat‑free mass index (FFMI), total body water percentage (TBW), and phase angle (PhA) were assessed. Between‑group differences and within‑child changes were analyzed using linear mixed effects models adjusted for age and sex.

**Results:**

At admission, children in the MUAC and Combined groups were younger and more stunted, with the Combined group showing the most severe deficits across anthropometry, FMI, and PhA. During treatment, the WHZ group exhibited the fastest recovery, with predominantly FFM accretion (82% of weight gain) and the largest increases in FFMI. In contrast, the MUAC group accumulated mainly FM (78% of weight gain) with minimal FFM gain and a decline in PhA, while the Combined group showed mixed FM and FFM recovery patterns.

**Conclusions:**

Children admitted for outpatient SAM treatment by MUAC, WHZ, or combined criteria appeared to follow different recovery trajectories despite meeting standard anthropometric discharge thresholds. Body‑composition and impedance indicators suggested heterogeneity in tissue gain patterns and vulnerability profiles across admission groups, particularly in the MUAC and Combined groups. While exploratory, these findings suggest that anthropometric recovery alone may not uniformly capture the full recovery pattern across SAM admission groups, warranting the need for longitudinal studies evaluating whether body-composition differences at discharge predict subsequent clinical outcomes and post-discharge vulnerability.

## Introduction

In 2024, an estimated 6.6% of children under five globally were affected by acute malnutrition and 1.9% by severe acute malnutrition (SAM), corresponding to 42.8 million and 12.2 million children, respectively, according to the United Nations Children’s Fund (UNICEF) [1]. Current World Health Organization (WHO) guidelines promote a public health approach emphasizing high program coverage, early identification, and differentiated care. Children with uncomplicated SAM—defined by anthropometric deficits without danger signs and/or bilateral edema—are treated as outpatients in the Outpatient Therapeutic Program (OTP) with ready‑to‑use therapeutic food (RUTF), while medically complicated cases require inpatient stabilization care. These pathways, introduced in 2013 and updated in 2023, emphasize prevention, early detection, outpatient management, and continuity of care following discharge [2].

For children aged 6–59 months, WHO and UNICEF recommend weight‑for‑height z‑score (WHZ <−3), mid‑upper arm circumference (MUAC <115 mm), and assessment of bilateral edema to identify SAM. In routine programs, MUAC and WHZ should be used together, as they correlate poorly and identify overlapping but distinct subsets of SAM cases [3–6]. Evidence indicates that an expanded MUAC‑only admission approach—often used as a temporary simplification in emergency or resource‑limited settings—may miss approximately 25% of SAM cases and misclassify an additional 20% as moderate acute malnutrition [7]. Accordingly, uncertainty persists regarding the biological and clinical differences between SAM groups defined by MUAC or WHZ alone [8], although re‑analyses consistently show substantial mortality risk among children identified by either criterion [9–11].

Discharge from SAM treatment remains largely guided by anthropometric thresholds (MUAC, WHZ, and edema resolution), despite their limited ability to reflect biological recovery or predict post‑discharge vulnerability. Findings from the 2024 multi‑country OptiDiag study indicate that children with WHZ‑only SAM may experience pronounced functional deficits (lower leptin levels, greater dehydration, and iron deficiency) comparable to or exceeding those seen in MUAC‑only cases, while children with combined MUAC and WHZ deficits show the greatest impairment at diagnosis. Treatment responses also differ, with WHZ‑only children often recovering more rapidly, whereas MUAC‑only children, particularly younger and more stunted children, tend to recover more slowly [12]. In line with the 2023 WHO guidelines calling for enhanced post‑exit monitoring, this highlights the need to better align discharge and referral criteria with physiological recovery, particularly given high relapse rates reported in sub‑Saharan Africa [13].

Fat mass (FM) and fat‑free mass (FFM) are independently associated with immune function and mortality risk [14,15], underscoring the potential value of body‑composition assessment, particularly via non‑invasive bioelectrical impedance analysis (BIA). Despite growing interest, associations between anthropometry and body composition remain insufficiently explored [14]. Evidence suggests that severe wasting involves deficits in both FM and FFM, whereas stunting—alone or combined with wasting—is associated with more heterogeneous FM patterns [14,16–20].

Treatment studies further support the relevance of body composition, as seen in important trials in Senegal and the Democratic Republic of Congo [16,17]. Phase angle, reflecting cell membrane integrity and body cell mass, is increasingly recognized as a functional biomarker of nutritional status and prognosis, with systematic reviews linking low PhA to undernutrition, complications, prolonged hospitalization, and mortality in pediatric populations [22–25].

Overall, while outpatient protocols effectively resolve wasting, deficits in FM, FFM, and linear growth frequently persist at anthropometric recovery, especially among younger and more stunted children. These findings highlight the potential value of integrating BIA‑derived insights into research on acute malnutrition to refine treatment approaches and support progress toward Sustainable Development Goal 2 on ending hunger and improving nutrition [21].

The hypothesis that distinct body‑composition recovery patterns among children admitted by MUAC only, WHZ only, or combined MUAC+WHZ criteria contribute to differential vulnerability warrants further investigation. Accordingly, this study aims to: (a) describe changes in anthropometry and body composition from admission to discharge among three cohorts of children aged 6–59 months with SAM; and (b) assess whether these groups differ in the extent and composition of nutritional recovery, with attention to fat deposition and lean mass accrual.

The study was terminated early in 2020 due to the COVID‑19 pandemic, preventing post‑discharge follow‑up.

## Materials and mMethods

### Participants and setting

This is a prospective cohort study of children with SAM aged 6–59 months enrolled and discharged as recovered from an outpatient therapeutic program. Children were admitted with a MUAC less than 115 mm (categorized as MUAC group), a WHZ < −3 z-score (categorized as WHZ group), or a combination of MUAC and WHZ < −3 z-score without medical complications (categorized as Combined group). They were analyzed longitudinally to observe and quantify the changes in body composition from admission to discharge from OTP as recovered children.

Inclusion criteria were: (i) children enrolled in outpatient treatment for SAM aged 6–59 months with a MUAC less than 115 mm, ii) children enrolled in outpatient treatment for SAM aged 6–59 months with a WHZ score < −3 z-score. Of children diagnosed with SAM, we excluded those with i) edema cases, ii) medical complications, iii) moderate acute malnutrition deteriorating to SAM and already under treatment with ready-to-use supplementary food (RUSF), and iv) those declining admission into the nutrition program or unable to provide consent.

BIA treats the body like it has stable water distribution and it assumes normal hydration and tissue properties. In edematous malnourished children, there is an increase of extracellular water, which makes the body appear more conductive. So, BIA misinterprets this as higher fat‑free mass (FFM), with a consequent overestimation of FFM and underestimation of fat mass (FM) [14,25]. This is the reason underlying the decision to exclude children with concurrent SAM and edema from the BIA analysis.

All participants were enrolled in outpatient therapeutic program (OTP) care and were managed as outpatients. The terms ‘admission’ and ‘discharge’ throughout the manuscript refer to admission to and discharge from the OTP nutrition program, not hospitalization. Children requiring inpatient care at any stage were referred to a Stabilization Center and were not included in the recovered cohort analysis. Medical complications excluded were defined in the national CMAM guidelines and were related to severe infections, such as severe pneumonia, severe malaria, sepsis, severe diarrhea with dehydration.

### Data collection

Data was collected at the Ministry of Health (MOH) primary health care facilities delivering community-based management of acute malnutrition (CMAM) services from 01/09/2019 to 15/04/2020 (recruitment period of the study) in Aweil Centre County, North Bahr el Ghazal State, South Sudan.

At each visit, enrolled children were given a RUTF ration around 200 kcal/kg/day. The dosage for all medicines and RUTF was given according to the South Sudan National Guidelines for the treatment of SAM [26]. Each child was discharged as recovered from the OTP and transferred to the TSFP when a MUAC of 115 mm or greater or a WHZ ≥ −3 z-score was obtained for 2 consecutive weekly outpatient visits, edema was absent, and the child was clinically well.

Detailed clinical management of SAM children after admission to the OTP as per national protocol included a standard course of amoxicillin to address minor subclinical infections, as well as albendazole and measles vaccination for all eligible unvaccinated children. Children diagnosed with malaria by rapid diagnostic testing were treated following national guidelines, typically involving combinations such as artesunate and amodiaquine. Participants were asked to return each week until recovery, which was defined as having attained a WHZ ≥ −3 for two consecutive visits for children with admission WHZ < −3, and MUAC ≥ 115 mm for two consecutive visits for children with admission MUAC < 115 mm. For children in the Combined group, as indicated by the South Sudan CMAM guidelines, either the MUAC threshold (≥ 115 mm for two consecutive visits) or the WHZ measurement threshold (WHZ ≥ −3 for two consecutive visits) was used as discharge criteria by the nutrition program staff, depending on whether the child was initially admitted to the OTP by MUAC or WHZ criteria. After recovery from OTP, the children were then transferred to the targeted supplementary feeding program (TSFP) where they were provided with RUSF (fixed dose: one sachet per day with a follow-up visit every 2 weeks) until reaching the exit criteria for 2 consecutive appointments (MUAC ≥125 mm or WHZ ≥ −2).

Admitted children were expected to meet discharge criteria from OTP and be transferred to TSFP within 8 weeks from initial admission. If the discharge criteria were not met within 8 weeks, the children were referred for further evaluation at hospital level and/or inpatient treatment at the Stabilization Center. ‘Defaulter’ was defined as child absent for 2 consecutive weekly appointments. ‘Non-responder’ was defined according to one the following situations: a) failure to gain any weight (non-edematous children) within 21 days from admission; b) weight loss for two successive visits (at any weekly appointment); weight below admission weight within 21 days from admission. After having ruled out problems related to the home environment and quality of treatment ‘non-responders’ were referred for medical investigation, including HIV testing and TB screening. No patients with HIV and/or TB were identified among the participants of the study.

MUAC, weight, height, triceps skinfold thickness (TSF), and bioelectrical impedance analysis (BIA) were measured on admission and when discharged as recovered from OTP. Variables measured included fat mass (FM), fat-free mass (FFM), total body water percentage (TBW), and phase angle (PhA), in addition to impedance. Length of stay and average daily weight gain were also calculated.

All staff were trained in anthropometric and body composition measurements. MUAC was measured to the nearest millimeter using a standard non-elastic MUAC tape (UNICEF “MUAC, Child 11.5, Red / PAC-50”). Weight was measured twice to the nearest 100 g using SECA® digital scales [16], which were calibrated daily. On admission and discharge, the child’s height was measured to the nearest millimeter using a standard pediatric height board, while the supine length was measured for children below 24 months of age. WHZ was then calculated using look-up tables in the above-mentioned CMAM guidelines.

Body composition was assessed through bioelectrical impedance analysis and triceps skinfold thickness. TSF was measured twice at the following four different time points: admission, after 3 visits, after 6 visits, and at discharge, using Harpenden® calipers to the nearest 0.2 mm, on a vertical fold of skin at the same location (i.e., the mid-point) on the upper arm as the MUAC measurement. The mean of the two TSF measurements was used in subsequent analyses. Calipers were checked and zeroed at each use and calibrated every month using a 10 mm metal calibration block. The same observer measured MUAC and TSF. BIA was assessed using BodyStat® 1500MD and measured at the same time points as TSF. Position quality for BIA was recorded and the measure repeated as appropriate. The device was checked according to manufacturer’s recommendations and measurement quality was verified at each assessment through electrode placement checks and evaluation of position quality.

This study was reported in accordance with the STROBE guidelines (Strengthening the Reporting of Observational Studies in Epidemiology) for cohort studies.

### Ethical considerations

The parents/guardians of all participants provided written informed consent to participate in the study. Ethics approval for this study was obtained from both the Ethic Review Board of the Ministry of Health of the Republic of South Sudan (MOH/ERB Ref: 14/2019) and the Institutional Review Board of the Seoul National University (SNU 25-12-054; IRB No. E2512/004–012).

Regarding written informed consent, individual consent from caregivers was obtained using a standardized information and consent form. The form explained the study’s purpose (assessing body composition and treatment response among children with severe acute malnutrition) and detailed the non-invasive procedures involved, including bioelectrical impedance analysis and skinfold measurements. It clarified that these measurements involve surface electrodes placed on the child’s hand and foot, harmless electrical current, and cleaning of the skin with alcohol wipes. It also specified eligibility criteria, confidentiality assurances, absence of direct medical benefit beyond standard care, and the lack of risk to the child. Participants were informed that refusal or withdrawal was permitted at any time without affecting access to care, and that their names were kept confidential and never included in study databases. Consent was documented through the caregiver’s signature or fingerprint and countersigned by trained clinic staff administering the consent process.

### Statistical analysis

Continuous variables are summarized as means and standard deviations (SD), and categorical variables as counts and percentages. Age is additionally presented as mode and range.

#### Baseline comparisons and assessment of attrition bias.

Baseline characteristics were compared between children included in the discharge analysis and those excluded (non‑recovered, defaulters, or referred) to assess potential selection and attrition bias. Each continuous baseline variable was compared between the two groups. Normality was assessed both visually using quantile-quantile (QQ) plots and analytically with the Shapiro–Wilk test, and homogeneity of variances was evaluated using Levene’s test. Because several variables were not normally distributed and group sizes were unequal, between-group comparisons were performed using the nonparametric Mann–Whitney U test. Effect sizes were expressed as rank-biserial correlations. Differences are reported as means or medians with 95% confidence intervals (95% CI), as appropriate. Effect sizes for non-parametric comparisons were reported as rank-biserial correlations (r), with values of approximately 0.10, 0.30, and 0.50 interpreted as small, moderate, and large effects, respectively. For categorical variables, effect sizes were reported as odds ratios (ORs) with 95% confidence intervals. Baseline comparisons were performed descriptively to evaluate whether children included in the discharge analysis differed systematically from those excluded in terms of age, sex, or baseline nutritional status. These comparisons were conducted for descriptive purposes rather than formal hypothesis testing; accordingly, p-values are presented for completeness but were not used to draw inferential conclusions, and no adjustment for multiple comparisons was applied. Any observed differences should therefore be interpreted as exploratory.

#### Longitudinal analysis.

The longitudinal analysis included two time points per child (admission and discharge). Longitudinal changes in body composition outcomes between admission and discharge were analyzed using linear mixed effects models to account for within‑subject correlation arising from repeated measurements. The model specification was:


$$Y_{ij}=\beta_{0}+\beta_{1}{\text{Time}}_{ij}+\beta_{2}{\text{Category}}_{i}+\beta_{3}\left({\text{Time}}_{ij}\times {\text{Category}}_{i}\right)\;\beta_{4}{\mathrm{Age}}_{i}+{ゲ}_{5}\mathrm{Sex}+b_{0i}+\epsilon_{ij}$$

where Yij denotes the outcome measure for child I (i = 1,…,N) at time point j (j = 1 for admission, j = 2 for discharge). Time was modeled as a binary within subject variable, whereas category, age, and sex were subject level covariates. The terms *b0i* represents a subject‑specific random effect (random‑intercept model), accounting for correlation between repeated measurements within the same child (random intercept model), and εij denotes the residual error. Models were fitted using restricted maximum likelihood estimation. Fixed effect estimates are reported as adjusted mean differences with 95% confidence intervals. For linear mixed-effects models, adjusted regression coefficients (β) and 95% confidence intervals are presented rather than standardized effect sizes because the coefficients are expressed in the original measurement units (e.g., kg/m^2^, %, z-scores), which provide greater clinical interpretability for longitudinal anthropometric and body-composition outcomes.

This approach provides a robust framework for analyzing repeated measures data while accounting for individual‑level heterogeneity [27].

#### Age subgroup analysis.

Age‑stratified analyses were conducted to explore whether changes in fat mass and fat‑free mass during recovery were influenced by age at admission. This analysis was motivated by the observed age distribution across admission categories, with children admitted based on MUAC being substantially younger than those admitted based on WHZ. Given known age‑related differences in body composition and growth dynamics in early childhood, age was considered both a potential confounder and effect modifier of post‑treatment body composition trajectories.

Statistical analysis was conducted using Stata® version 14 (StataCorp, College Station, TX, USA) and R® version 3.6.3.

Based on the WHO child growth standard [28], anthropometric data was transformed into a standard deviation score for comparison after being matched with age and sex. Triceps skinfold thickness z‑scores (TSFZ) were calculated using WHO references [28], allowing standardization by age and sex.

Intermediate follow‑up measurements (e.g., week 3 or week 6) were sporadically collected as part of routine program monitoring: as these data were incomplete, they were not included in the present analysis.

We also analyzed differences in anthropometry and body composition measurements between admission and discharge of the malnourished children discharged as recovered (paired cohort = 127 children).

The primary outcomes were changes in FMI and FFMI between admission and discharge. Secondary outcomes included changes in FM, FFM, TBW percentage, phase angle, TSFZ, MUAC, WHZ, WAZ, and HAZ.

Missing data was minimal (only two TSF at admission and one TBW at discharge measurement missing). Where measurements were missing, those observations were excluded from analyses on a variable-specific basis.

Formal inter-observer reliability statistics were not collected.

## Results

### Participant flow. Descriptive statistics, baseline anthropometry and BIA values at admission

**Fig 1:**
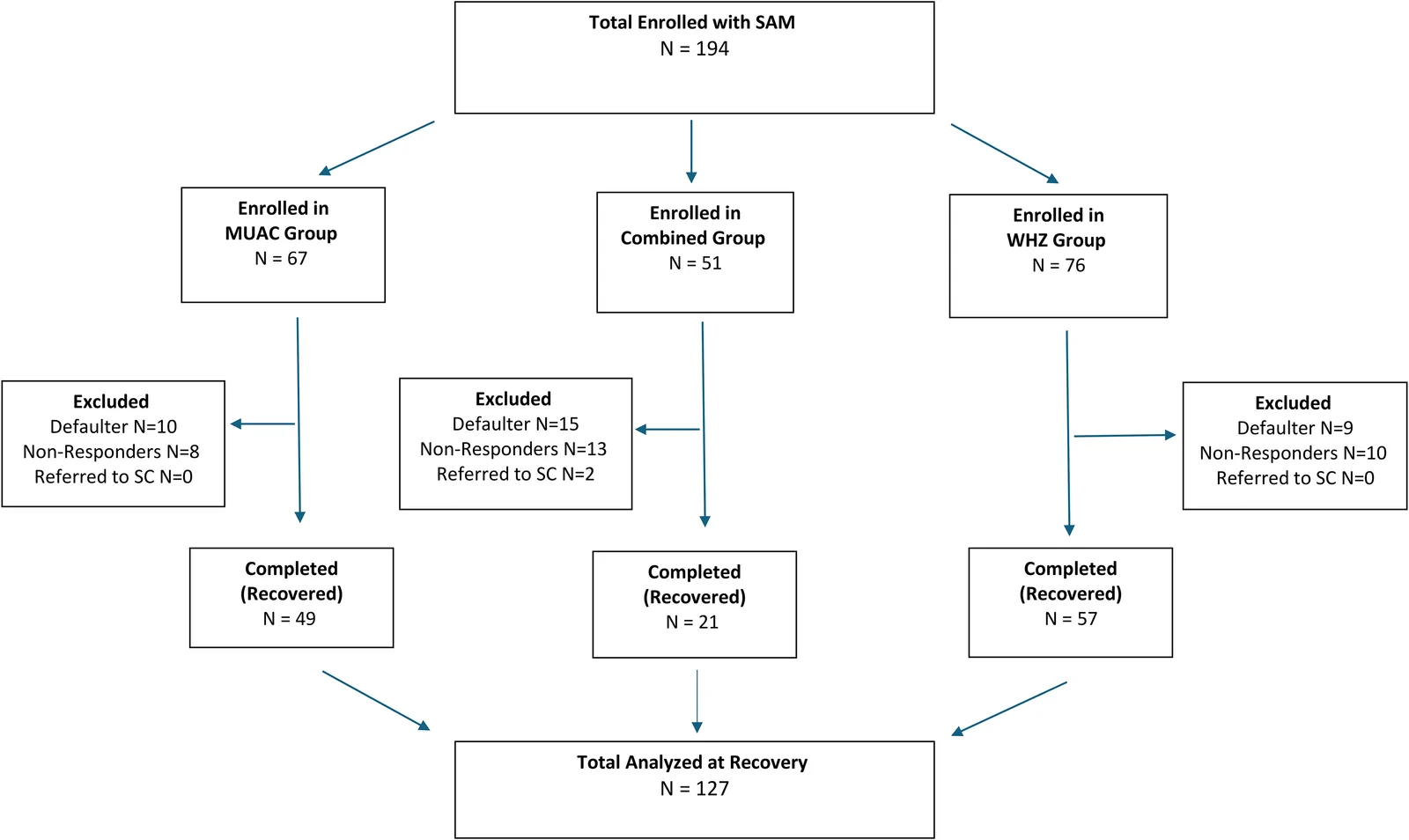
STROBE flow diagram showing participant enrolment, follow‑up outcomes, and inclusion in the final analysis. A total of 194 children admitted with severe acute malnutrition (SAM) were enrolled and classified at admission into MUAC‑only, WHZ‑only, or combined MUAC+WHZ groups. During follow‑up, children were classified as recovered, defaulters, non‑responders, or referred to a stabilization center. Only children classified as recovered were included in the discharge analysis. *SAM, severe acute malnutrition; MUAC, mid-upper arm circumference; WHZ, weight-for-height z-score; SC, stabilization center.*

A total of 194 children aged 6–59 months were enrolled at admission: 67 met SAM admission criteria by MUAC only (MUAC group), 51 by a combination of MUAC+WHZ (Combined group), and 76 by WHZ only (WHZ group) (Fig 1, Table 1). Of these, 127 were followed to recovery at discharge (MUAC group: n = 49; Combined group: n = 21; WHZ group: n = 57).

**Table 1:** Baseline characteristics of recovered children included (n = 127) in the study discharge analysis compared with excluded children (n = 67), stratified by severe acute malnutrition (SAM) admission criteria.

		MUAC Group				Combined Group				WHZ Group			
		N	Value	p-value Chi2	Effect Size	N	Value	p-value Chi2	Effect Size	N	Value	p-value Chi2	Effect Size
Sex, female (N)	**Included**	**49**	32	0.465	OR 1.50 (95% CI 0.43–5.16)	**21**	9	0.788	OR 0.857 (95% CI 0.24–3.02)	**57**	13	0.755	OR 0.827 (95% CI 0.22–3.50)
	**Excluded**	**18**	10			**30**	14			**19**	5		
				**p-value** **Rank Sum**	**Rank-biserial ‘r’** **value**			**p-value** **Rank Sum**	**Rank-biserial ‘r’ value**			**p-value** **Rank Sum**	**Rank-biserial ‘r’ value**
Age in months (Mean and 95% CI)	**Included**		12.1 (9.6 −14.5)	0.897	0.02		14.9 (10.1-19.8)	0.961	−0.01		20.5 (17.6-23.4)	0.431	−0.09
	**Excluded**		11.1 (7.6-14.6)				15.8 (11.4-20.1)				17.3 (13.6-20.9)		
Height, cm (Mean and 95% CI)	**Included**		67.0 (65.1-68.9)	0.794	0.03		69.1 (64.9-73.3)	0.619	0.07		77.2 (72.1-79.6)	0.991	−0.01
	**Excluded**		67.3 (64.4-70.1)				70.3 (66-6-74.0)				75.9 (74.9-79.5)		
Weight, Kg (Mean and 95% CI)	**Included**		6.01 (5.70-6.31)	0.501	0.08		5.87 (5.09-6.66)	0.592	0.08		7.58 (7.18-7.97)	0.737	−0.04
	**Excluded**		6.27 (5.75-6.78)				6.01 (5.43-6.59)				7.20 (6.57-7.82)		
MUAC, cm (Mean and 95% CI)	**Included**		11.03 (10.92-11.15)	0.169	0.17		10.68 (10.35-11.01)	0.544	0.09		12.07 (11.97-12.17)	0.375	0.10
	**Excluded**		11.19 (11.09-11.29)				10.82 (10.58-11.06)				11.88 (11.33-12.42)		
WHZ (Mean and 95% CI)	**Included**		−2.70 (−3.02 −2.38)	0.365	−0.11		−4.00 (−4.42 −3.58)	0.297	−0.15		−3.40 (−3.56 −3.25)	**0.027**	**−0.25**
	**Excluded**		−2.58 (−3.14 −2.02)				−4.17 (−4.45 −3.88)				−3.73 (−4.17 −3.29)		
WAZ (Mean and 95% CI)	**Included**		−3.41 (−3.75 −3.08)	0.312	0.12		−4.55 (−5.07 −4.02)	0.473	0.10		−3.39 (−3.64 −3.14)	0.443	0.09
	**Excluded**		−2.99 (−3.41 −2.56)				−4.38 (−4.84 −3.91)				−3.42 (−4.16 −2.70)		
HAZ (Mean and 95% CI)	**Included**		−2.33 (−2.87 −1.79)	0.373	0.11		−2.88 (−3.50 −2.26)	0.228	0.17		−1.89 (−2.30 −1.49)	0.09	0.19
	**Excluded**		−1.88 (−2.59 −1.17)				−2.30 (−2.93 −1.67)				−1.38 (−2.16 −0.60)		
TSFZ (Mean and 95% CI)	**Included**		−2.84 (−3.10 −2.57)	0.408	0.11		−3.56 (−4.14 −2.98)	0.234	0.17		−1.69 (−1.98 −1.41)	0.971	0.01
	**Excluded**		−2.62 (−3.02 −2.21)				−3.12 (−3.59 −2.67)				−1.65 (−2.34 −0.97)		
FMI Kg/m^2^ (Mean and 95% CI)	**Included**		4.02 (3.61-4.42)	0.221	0.15		3.46 (2.97-3.95)	0.985	−0.01		4.02 (3.71-4.33)	0.810	−0.03
	**Excluded**		4.43 (3.62-5.25)				3.65 (2.92-4.37)				4.09 (3.59-4.58)		
FFMI Kg/m^2^ (Mean and 95% CI)	**Included**		9.32 (8.90-9.73)	0.777	0.03		8.63 (8.24-9.02)	1.00	0		8.63 (8.30-8.96)	0.418	−0.09
	**Excluded**		9.28 (8.59-9.97)				8.64 (8.21-9.08)				8.34 (7.88-8.80)		
TBW %, (Mean and 95% CI)	**Included**		54.97 (52.71-57.23)	0.258	0.14		56.38 (53.61-59.14)	0.878	0.02		53.31 (51.40-55.23)	0.692	0.05
	**Excluded**		53.28 (49.13-57.43)				56.22 (53.74-58.70)				53.07 (50.55-55.59)		
Impedance Ω (Mean and 95% CI)	**Included**		952.6 (911.2-994)	0.734	0.04		1006.1 (955.2-1057.1)	0.521	0.09		1034.5 (996.7-1072.2)	0.573	0.06
	**Excluded**		959.6 (887.4-1031.7)				1011.3 (968.2-1054.5)				1061.4 (1008.9-1114)		
PhA (Mean and 95% CI)	**Included**		2.78 (2.43-3.12)	0.466	0.09		2.19 (1.94-2.43)	0.618	0.07		2.87 (2.43-3.31)	0.410	−0.10
	**Excluded**		3.21 (2.32-4.10)				2.21 (1.87-2.56)				2.74 (2.04-3.44)		

Baseline demographic, anthropometric, and body composition characteristics are shown for included (127) and excluded (67) children from the discharge analysis within each SAM admission group (MUAC, WHZ, and combined MUAC + WHZ). Continuous variables are presented as mean and 95% confidence interval and were compared between included and excluded children using independent‑samples t tests or Wilcoxon rank‑sum tests, as appropriate. Categorical variables are presented as counts and were compared using chi‑square tests. Effect sizes are reported as odds ratios (OR) with 95% confidence intervals for categorical variables and rank‑biserial correlation coefficients (r) for non‑parametric comparisons.

### Baseline comparison between recovered and non‑recovered children

Among 194 enrolled children, 127 were classified as recovered and included in the discharge analysis, while 67 were excluded due to non-recovery outcomes. Baseline characteristics were compared between recovered and non-recovered children within each admission group (MUAC, WHZ, and Combined) to assess potential attrition or survivor bias.

Overall, recovered and non-recovered children showed largely comparable baseline demographic, anthropometric, and body composition characteristics across groups. No meaningful differences were observed for age, sex, height, weight, MUAC, WAZ, HAZ, TSFZ, FMI, FFMI, TBW percentage, impedance, or PhA, with small effect sizes (r generally <0.20) and non-significant p values.

A statistically significant difference was observed only for baseline WHZ values within the WHZ group. In this group, recovered children had a higher WHZ at admission compared with non-recovered children, corresponding to a small to moderate effect size (rank biserial r = −0.25; p = 0.027). No corresponding differences were detected within the MUAC or Combined admission groups.

### Characteristics of the children with SAM on admission (194 children)

On admission, the WHZ group was the oldest (mean age 19.7 ± 10.3 months) compared with the Combined (15.4 ± 11.2 months) and MUAC (11.8 ± 8.1 months) groups; the sex distribution also differed, with girls comprising 62.7% in MUAC, 45.1% in Combined, and 23.7% in WHZ. At discharge (recovered cohort), mean ages were 14.2 ± 9.4, 16.4 ± 10.6 and 22.0 ± 11.2 months for MUAC, Combined and WHZ, respectively, and girls represented 65.3%, 42.9% and 22.8% in the same groups (Table 1).

At admission, children in the WHZ group were older, taller, and heavier than those in the MUAC and Combined groups, whereas children in the Combined group exhibited the lowest triceps skinfold thickness and the most severe anthropometric deficits (S1 Table).

Body-composition profiles were broadly comparable across the three admission groups in terms of overall fat proportion and total body water percentage. Nevertheless, children in the Combined group tended to present less favorable body-composition indicators, with lower fat mass index (FMI), lower fat-free mass index (FFMI), and lower phase angle values than children admitted by MUAC or WHZ alone. These findings are consistent with the more severe anthropometric deficits observed in this group at enrollment (S1 Table).

Adjusted baseline analyses indicated that children in the Combined group presented the most compromised nutritional status at admission, with poorer body-composition and anthropometric indicators than children admitted by either MUAC or WHZ alone. Children in the WHZ group were generally taller, less stunted, and had greater triceps skinfold thickness, while both the MUAC and WHZ groups showed more favorable anthropometric and bioimpedance profiles than the Combined group (Tables 2-6).

**Table 2:** Descriptive admission‑to‑discharge characteristics of the paired cohort of children with severe acute malnutrition (SAM) who recovered (n = 127).

		MUAC Group	Combined Group	WHZ Group
***Total of the paired cohort***	**127**	**49**	**21**	**57**
***Sex, female (N and %)***		32 (65.3%)	9 (42.9%)	13 (22.8%)
***Age in months,*** ***Mean ± SD (95% CI), p-value***	Admission	12.08 **±** 8.52 (9.67–14.49)	14.90 **±** 10.74 (10.27–19.54)	20.51 **±** 10.98 (17.63–23.39)
	Discharge	14.24 **±** 9.42 (11.58 −16.92)	16.38 **±** 10.60 (11.80–20.96)	21.98 **±** 11.16 (19.05–24.91)
	p-value	0.236	0.656	0.478
***Age in months (Range), Mode***	Admission	(6-36), 6	(6-48), 6	(6-54), 24
	Discharge	(7-44), 7	(7-50), 8	(7-55),15
***Height, cm,*** ***Mean± SD (95% CI), p-value***	Admission	67.0 **±** 6.6 (65.16–68.87)	69.1 **±** 9.30 (65.08–73.11)	77.2 **±** 8.6 (74.96–79.49)
	Discharge	68.1 **±** 6.1 (66.35–69.81)	70.8 **±** 8.6 (67.08–74.53)	78.1 **±** 8.3 (75.87–80.23)
	p-value	0.408	0.539	0.601
***Weight, Kg,*** ***Mean ± SD (95% CI)), p-value***	Admission	6.01 **±** 1.07 (5.70–6.30)	5.87 **±** 1.72 (5.13–6.62)	7.58 **±** 1.4 (7.19–7.97)
	Discharge	6.74 **±** 1.14 (6.42–7.06)	6.73 **±** 1.64 (6.02–7.44)	8.29 **±** 1.44 (7.91–8.66)
	p-value	**0.001**	0.106	**0.011**
**MUAC, cm,** **Mean ± SD (95% CI), p-value**	Admission	11.03 **±** 0.48 (10.92 −11.15)	10.68 **±** 0.3 (10.37–10.99)	12.07 **±** 0.36 (11.98–12.17)
	Discharge	11.70 **±** 0.51 (11.56–11.85)	11.67 **±** 0.38 (11.51–11.84)	12.32 **±** 0.55 (12.17–12.46)
	p-value	**<0.001**	**<0.001**	**0.006**
***TSF, mm,*** ***Mean ± SD (95% CI)), p-value***	Admission	4.99 **±** 0.85 (4.75–5.23)	4.31 **±** 0.85 (3.94–4.68)	5.5 **±** 1.15 (5.55–6.15)
	Discharge	5.80 **±** 1.18 (5.47 - 6.14)	5.73 **±** 0.79 (5.39–6.08)	6.28 **±** 1.20 (5.97–6.60)
	p-value	**0.001**	**<0.001**	0.051
***HAZ,*** ***Mean ± SD (95% CI)), p-value***	Admission	−2.32 **±** 1.86 (−2.85 −1.80)	−2.88 **±** 1.37 (−3.47 −2.29)	−1.90 **±** 1.54 (−2.30 −1.49)
	Discharge	−2.74 **±** 1.66 (−3.21 −2.27)	−2.84 **±** 1.19 (−3.36 −2.33)	−2.02 **±** 1.59 (−2.43 −1.61)
	p-value	0.245	0.928	0.677
***WAZ,*** ***Mean ± SD ((95% CI)), p-value***	Admission	−3.41 **±** 1.17 (−3.75 −3.08)	−4.55 **±** 1.14 (−5.04 −4.05)	−3.39 **±** 0.94 (−3.64 −3.14)
	Discharge	−2.91 **±** 1.17 (−3.24 −2.58)	−3.64 **±** 0.87 (−4.01 −3.25)	−2.81 **±** 0.97 (−3.07 −2.56)
	p-value	**0.036**	**0.006**	**0.001**
***WHZ,*** ***Mean ± SD (95% CI), p-value***	Admission	−2.70 **±** 1.11 (−3.01 −2.38)	−4.00 **±** 0.92 (−4.40 −3.60)	−3.40 **±** 0.59 (−3.56 −3.25)
	Discharge	−1.71 **±** 1.00 (−1.99 −1.43)	−2.81 **±** 0.72 (−3.12 −2.50)	−2.45 ± 0.68 (−2.63 −2.28)
	p-value	**<0.001**	**<0.001**	**<0.001**
***TSFZ,*** ***Mean ± SD (95% CI), p-value***	Admission	−2.84 **±** 0.90) (−3.09 −2.58)	−3.56 **±** 1.27) (−4.11 −3.01)	−1.69 **±** 1.06 (−1.98 −1.41)
	Discharge	−1.93 **±** 1.01) (−2.22 −1.64)	−1.85 **±** 0.78) (−2.19 −1.51)	−1.29 **±** 1.03 (−1.56 −1.01)
	p-value	**<0.001**	**<0.001**	**0.040**
***Length of Stay (LoS), days***		46.5	51.9	30.6
***Average Weight Gain (AWG) g/Kg/day***		2.62	2.81	3.06
***FFM, kg,*** ***Mean ± SD (95% CI), p-value***	Admission	4.20 **±** 0.95 (3.93–4.47)	4.17 **±** 1.19 (3.65 −4.68)	5.16 **±** 1.18 4.85–5.46)
	Discharge	4.36 **±** 0.82 (4.13–4.59)	4.66 **±** 1.07 (4.20–5.12)	5.74 **±** 1.29 (5.40–6.07)
	p-value	0.379	0.167	**0.014**
***FM, Kg,*** ***Mean ± SD (95% CI), p-value***	Admission	1.81 **±** 0.72 (1.60 −2.01)	1.70 **±** 0.73 (1.39–2.02)	2.42 **±** 0.88 (2.19–2.65)
	Discharge	2.38 **±** 0.65 (2.20–2.56)	2.07 **±** 0.82 (1.72–2.43)	2.55 **±** 0.91 (2.31–2.79)
	p-value	**<0.001**	0.134	0.442
Fat % [FM/Weight *100]	Admission	30.1%	29.0%	31.9%
	Discharge	35.3%	30.8%	30.8%
***FFMI, Kg/m*** ^***2,***^ ***Mean ± SD (95% CI), p-value***	Admission	9.31 **±** 0.21 (8.91–9.73)	8.63 **±** 0.19 (8.26–9.00)	8.63 **±** 1.24 (8.31–8.96)
	Discharge	9.38 **±** 1.12	9.26 **±** 0.23	9.38 **±** 1.43
	p-value	0.798	**0.036**	**0.004**
***FMI, Kg/m*** ^***2***^ ***Mean ± SD (95% CI), p-value***	Admission	4.02 **±** 1.41 (3.62–4.42)	3.46 **±** 1.08 (2.99–3.92)	4.03 **±** 1.18 (3.72–4.34)
	Discharge	5.11 **±** 1.03 (4.82–5.41)	4.04 **±** 0.97 (3.62–4.46)	4.21 **±** 1.46 (3.83–4.59)
	p-value	**<0.001**	0.073	0.461
***TBW %,*** ***Mean ± SD (95% CI), p-value***	Admission	54.97 **±** 7.88 (52.74–57.20)	56.38 **±** 6.07 (53.75–59.00)	53.31 **±** 7.22 (51.42–55.21)
	Discharge	50.82 **±** 5.39 (49.29 - 52.37)	55.85 **±** 9.09 (51.93–59.78)	54.28 **±** 7.63 (52.28–56.28)
	p-value	**0.003**	0.827	0.489
***Impedance Ω,*** ***Mean ± SD (95% CI), p-value***	Admission	952.59 **±** 144.27 (911.80–993.38)	1006.14 **±** 111.86 (957.84–1054.45)	1034.47 **±** 142.21 (997.20–1071.75)
	Discharge	946.06 **±** 136.38 (907.51 −984.62)	942.38 **±** 138.59 (882.53–1002.23)	939.96 **±** 139.31 (903.45 −976.48)
	p-value	0.818	0.108	**<0.001**
***PhA,*** ***Mean ± SD (95% CI), p-value***	Admission	2.8 **±** 1.2 (2.4–3.1)	2.2 **±** 0.5 (2.0–2.4)	2.9 **±** 1.7 (2.4–3.3)
	Discharge	2.3 **±** 0.8 (2.1–2.5)	2.7 **±** 1.5 (2.1 −3.4)	3.0 **±** 2.0 (2.5–3.5)
	p-value	**0.024**	0.107	0.731

The table presents data for the 127 children with SAM who were assessed both at admission and at discharge after achieving nutritional recovery, allowing paired comparisons over the course of treatment. Anthropometric measurements and body composition parameters reflect changes occurring between admission and discharge following nutritional recovery. All values are reported as mean ± SD; 95% confidence intervals are shown in parentheses where indicated. They reflect unadjusted paired observations and are presented for descriptive purposes.

SAM, severe acute malnutrition; OTP, outpatient therapeutic program; SD, standard deviation; CI, Confidence interval; MUAC, Mid-upper arm circumference; WHZ, Weight-for-height z score; TSF, triceps skinfold thickness; TSFZ, triceps skinfold thickness z score; HAZ, Height-for-age z score; WAZ, Weight-for-age z score; TBW, total body water; FM, fat mass; FMI, fat mass index; FFM, fat free mass; FFMI, fat free mass index; PhA, phase angle.

**Table 3:** Total weight gain and relative contributions of fat mass (FM) and fat‑free mass (FFM) during nutritional treatment in the paired cohort of children (n = 127) with severe acute malnutrition (SAM) who recovered following outpatient therapeutic program (OTP) care.

	*MUAC Group*	*Combined Group*	*WHZ Group*
***Weight Gain (in kg)***	0.73	0.86	0.71
***Average weight Gain (g/kg/day)***	2.62	2.81	3.06
***FM Gain (in kg)***	0.57	0.37	0.13
***% FM Contribution to the Weight Gain***	78.1%	43.0%	18.3%
***FFM Gain (in kg)***	0.16	0.49	0.58
***% FFM Contribution to the Weight Gain***	21.9%	57.0%	81.7%

The table summarizes changes in body weight and body composition among the 127 children with SAM who had paired measurements at admission and at recovery. Absolute gains in body weight, FM, and FFM are reported in kilograms, while average weight gain is expressed as grams per kilogram per day. The percentage contribution of FM and FFM to total weight gain was calculated for each group (MUAC, WHZ, Combined) based on paired admission‑to‑discharge differences. Values are not adjusted for age or sex.

SAM, severe acute malnutrition; OTP, outpatient therapeutic program; WHZ, Weight for height z score; MUAC, Mid-upper arm circumference; FM, fat mass; FFM, fat free mass.

**Table 4:** Changes in body fat proportion, fat mass index (FMI), fat‑free mass index (FFMI), and mid‑upper arm circumference (MUAC) velocity during nutritional rehabilitation in the paired cohort of children (n = 127) with severe acute malnutrition (SAM).

	*MUAC Group*	*Combined Group*	*WHZ Group*
Change in FM % [FM/Weight *100] (SD)	5.2	1.8	−1.1
***Change FMI, Kg/m***^***2***^ ***(SD)***	1.09 (1.42)	0.58 (1.06)	0.18 (1.65)
***Change FFMI, Kg/m***^***2***^ ***(SD)***	0.24 (1.25)	0.63 (1.04)	0.74 (1.79)
***MUAC, mm/day***	1.44	1.90	0.65

The table presents paired admission‑to‑discharge changes among the 127 children with SAM who recovered following outpatient therapeutic program (OTP) treatment. Changes in fat mass percentage, FMI, and FFMI are reported as mean differences between admission and recovery. MUAC velocity is expressed as millimeters gained per day over the treatment period. This table presents unadjusted mean changes; adjusted estimates accounting for age, sex, and repeated measurements are reported separately in Tables 9–10.

SAM, severe acute malnutrition; OTP, outpatient therapeutic program; WHZ, Weight-for-height z score; MUAC, Mid-upper arm circumference; FM, fat mass; FMI, fat mass index; FFMI, fat free mass index.

**Table 5:** Linear mixed effects models of baseline anthropometric and body‑composition outcomes at admission (n = 194), with the MUAC group as reference. Coefficients are shown with 95% confidence intervals and p‑values; models adjusted for age and sex.

	FMI		FFMI		TBW		PhA		WHZ		WAZ		HAZ		TSFZ	
	Coeff (95% CI)	p- value	Coeff (95% CI)	p- value	Coeff (95% CI)	p- value	Coeff (95% CI)	p- value	Coeff (95% CI)	p- value	Coeff (95% CI)	p- value	Coeff (95% CI)	p- value	Coeff (95% CI)	p- value
Intercept	4.175		9.209		54.114		2.885		2.746		−3.443		−2.483		−2.694	
WHZ Group	−0.179 (−0.655 0.297)	0.461	−0.567 (−0.998 −0.137)	**0.010**	−0.500 (−3.019 2.019)	0.697	−0.047 (−0.560 0.466)	0.857	−0.666 (−0.972 −0.359)	**<0.001**	0.174 (−0.220 0.567)	0.387	0.965 (0.406 1.523)	**0.001**	0.933 (0.552 1.315)	**<0.001**
Combined Group	−0.602 (−1.092 −0.112)	**0.016**	−0.587 (−1.031 −0.143)	**0.010**	2.115 (−0.481 4.712)	0.110	−0.684 (−1.206 −0.162)	**0.010**	−1.363 (−1.678 −1.050)	**<0.001**	−1.026 (−1.425 −0.627)	**<0.001**	−0.092 (−0.658 0.473)	0.748	−0.608 (−0.998 −0.219)	**0.002**
Gender Female	−0.328	**0.066**	−0.111	0.482	0.762	0.415	0.179	0.369	0.221	0.059	0.259	0.103	0.492	**0.030**	0.027	0.856
Age	−0.004	0.584	−0.028	**<0.001**	−0.059	0.177	0.007	0.461	−0.009	0.119	−0.022	**0.004**	−0.042	**<0.001**	0.018	**0.012**

FMI, fat mass index; FFMI, fat free mass index; TBW, total body water; WHZ, Weight-for-height z-score; WAZ, weight-for-age z-score; HAZ, height-for-age z-score; TSFZ, triceps skinfold thickness – score.

**Table 6:** Linear mixed effects models of baseline anthropometric and body‑composition outcomes at admission (n = 194), with the Combined group as reference. Coefficients are shown with 95% confidence intervals and p‑values; models adjusted for age and sex.

	FMI		FFMI		TBW		PhA		WHZ		WAZ		HAZ		TSFZ	
	Coeff (95% CI)	p- value	Coeff (95% CI)	p- value	Coeff (95% CI)	p- value	Coeff (95% CI)	p- value	Coeff (95% CI)	p- value	Coeff (95% CI)	p- value	Coeff (95% CI)	p- value	Coeff (95% CI)	p- value
Intercept	3.573		8.622		56.230		−2.200		−4.110		−4.469		−2.576		−3.302	
MUAC Group	−0.602 (0.112 1.092)	**0.016**	−0.587 (0.143 1.031)	**0.010**	−2.115 (−4.712 0.481)	0.110	0.684 (0.161 1.206)	**0.01**	1.363 (1.050 1.678)	**<0.001**	1.026 (0.627 1.425)	**<0.001**	−0.092 (−0.473 0.658)	0.748	0.608 (0.219 0.998)	**0.001**
WHZ Group	0.423 (−0.058 0.904)	0.085	0.019 (−0.416 0.456)	0.930	−2.616 (−5.166 −0.065)	**0.044**	0.637 (0.121 1.153)	**0.016**	0.698 (0.389 1.006)	**<0.001**	1.200 (0.807 1.592)	**<0.001**	1.057 (0.501 1.614)	**<0.001**	1.542 (1.158 1.925)	**<0.001**
Gender Female	−0.328	**0.066**	−0.111	0.482	0.762	0.415	0.179	0.369	0.221	0.059	0.259	0.103	0.492	**0.030**	0.027	0.856
Age	−0.004	0.584	−0.028	**<0.001**	−0.059	0.177	0.007	0.461	−0.009	0.119	−0.022	**0.004**	−0.042	**<0.001**	0.018	**0.012**

FMI, fat mass index; FFMI, fat free mass index; MUAC, Mid-upper arm circumference; TBW, total body water; WHZ, Weight-for-height z-score; WAZ, weight-for-age z-score; HAZ, height-for-age z-score; TSFZ, triceps skinfold thickness z-score.

### Treatment exposure and growth during OTP

Length of stay by group was 51.9 days, for children in the Combined group; 46.5 days for MUAC group; 30.6 days for children in the WHZ group. Children in the MUAC group gained an average of 2.61 g/kg/day compared to 2.81 g/kg/day in the Combined group and 3.06 g/kg/day in the WHZ group (Table 2). MUAC velocity (mm/day) among recovered children was greatest in Combined (1.90), followed by MUAC (1.44) and WHZ (0.65) groups (Table 4).

### Anthropometry and BIA values among recovered children from OTP. Within-child changes (paired cohort, n = 127)

Table 2 summarizes anthropometric and body-composition measurements at admission and discharge among the 127 children who recovered from SAM. All admission groups experienced improvements in weight and MUAC over the course of treatment. Nevertheless, differences between groups persisted at discharge. Children admitted by MUAC alone achieved the most favorable WHZ and WAZ values at recovery, whereas children in the Combined group continued to show the poorest anthropometric status. Linear growth deficits also remained evident across all groups, with stunting being particularly pronounced among children admitted by MUAC and those meeting both MUAC and WHZ criteria. These findings suggest that anthropometric recovery did not fully eliminate the baseline differences observed between admission groups (Table 2).

**Fig 2:**
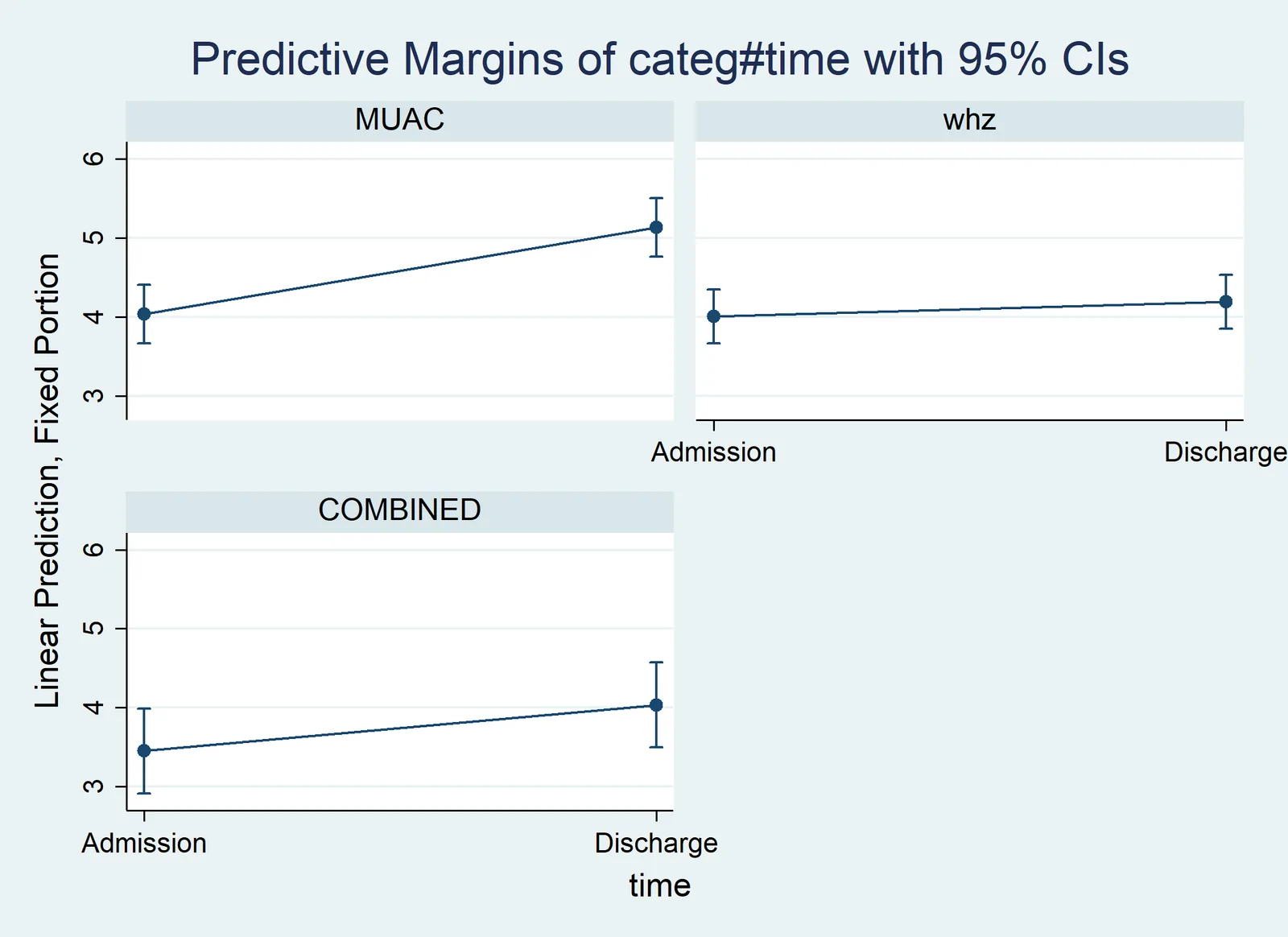
Predicted margins from linear mixed effects models of FMI. Predicted margins of FMI at admission and discharge by admission group. Estimates are from linear mixed effects models accounting for repeated measurements within individuals (n = 127), with predicted values shown with 95% confidence intervals. FMI, fat mass index; SAM, severe acute malnutrition; OTP, outpatient therapeutic program; MUAC, mid‑upper arm circumference; WHZ, weight‑for‑height z‑score.

**Fig 3:**
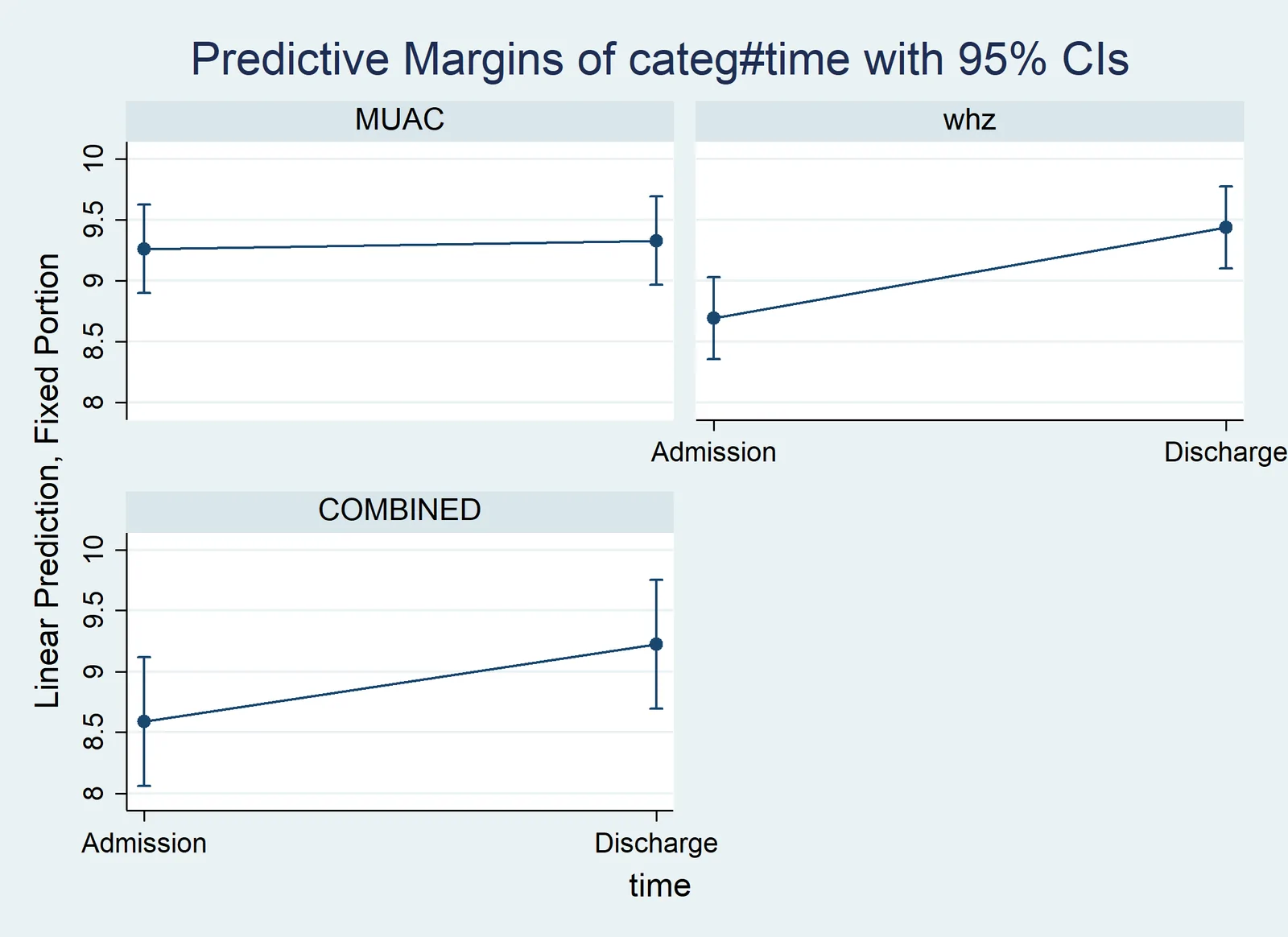
Predicted margins from linear mixed effects models of FFMI. Predicted margins of FFMI at admission and discharge by admission group. Estimates are from linear mixed effects models accounting for repeated measurements within individuals (n = 127), with predicted values shown with 95% confidence intervals. FFMI, fat free mass index; SAM, severe acute malnutrition; OTP, outpatient therapeutic program; MUAC, mid‑upper arm circumference; WHZ, weight‑for‑height z‑score.

Body composition responses from admission to discharge differed by admission group. FMI increased the most in the children of the MUAC group (+1.09 ± 1.42 kg/m^2^), moderately in the Combined group (+0.58 ± 1.06 kg/m^2^), and minimally in the WHZ group (+0.18 ± 1.65 kg/m^2^); the fat component percentage of the total body weight rose mostly in the MUAC group (by +5.2 points). (Fig 2, S1 Fig, Table 4). FFMI increased in all groups, with larger gains in the WHZ group (+0.74 ± 1.79 kg/m^2^) and the smaller gain in the MUAC group (+0.24 ± 1.25 kg/m^2^ (Fig 3, S2 Fig, Table 4). Consequently, the relative contribution of fat and lean tissue to weight gain varied substantially across groups, indicating distinct tissue-recovery trajectories despite similar anthropometric recovery outcomes.

**Fig 4:**
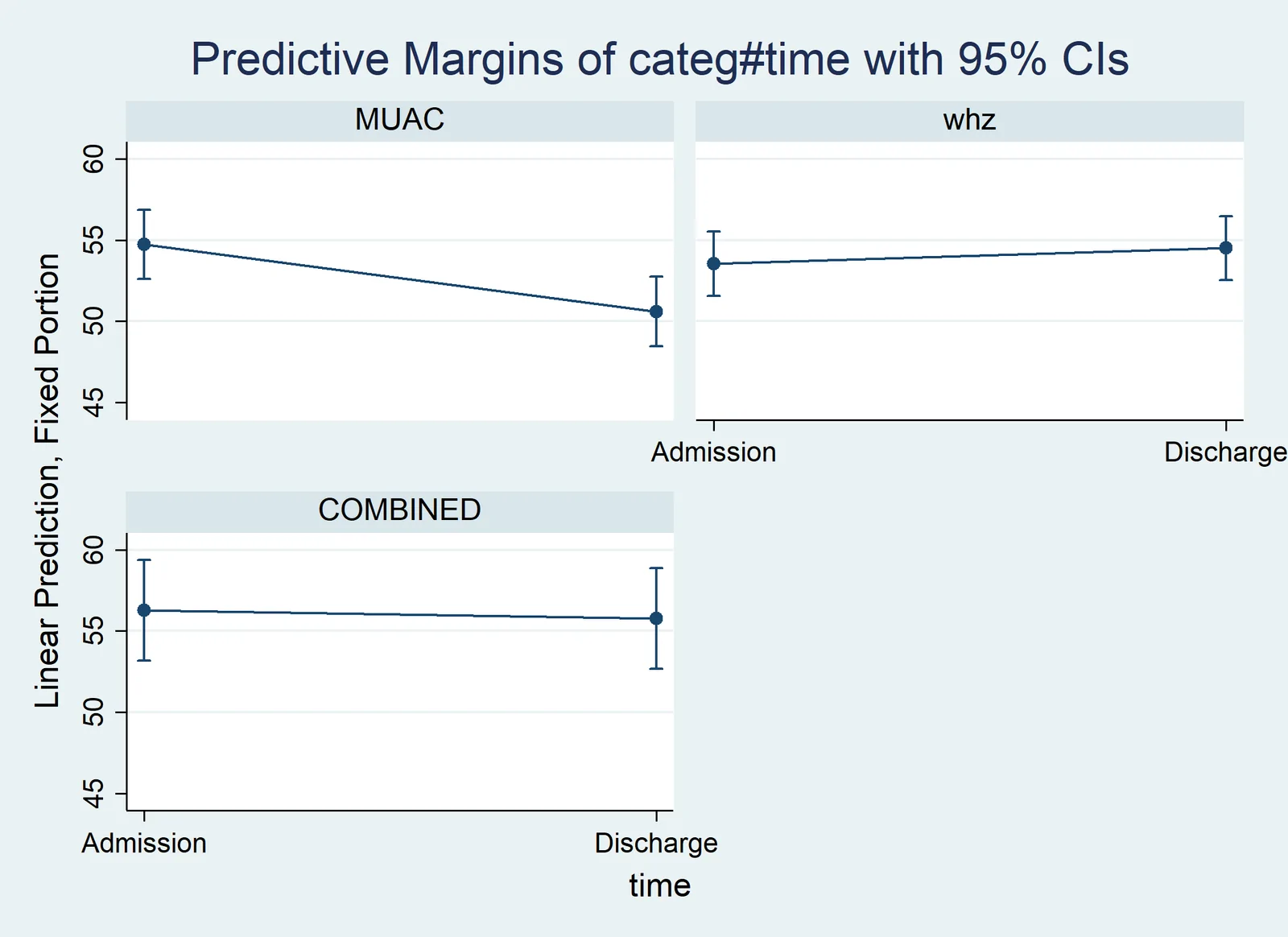
Predicted margins from linear mixed effects models of TBW. Predicted margins of TBW at admission and discharge by admission group. Estimates are from linear mixed effects models accounting for repeated measurements within individuals (n = 127), with predicted values shown with 95% confidence intervals. TBW, total body water percentage; SAM, severe acute malnutrition; OTP, outpatient therapeutic program; MUAC, mid‑upper arm circumference; WHZ, weight‑for‑height z‑score.

**Fig 5:**
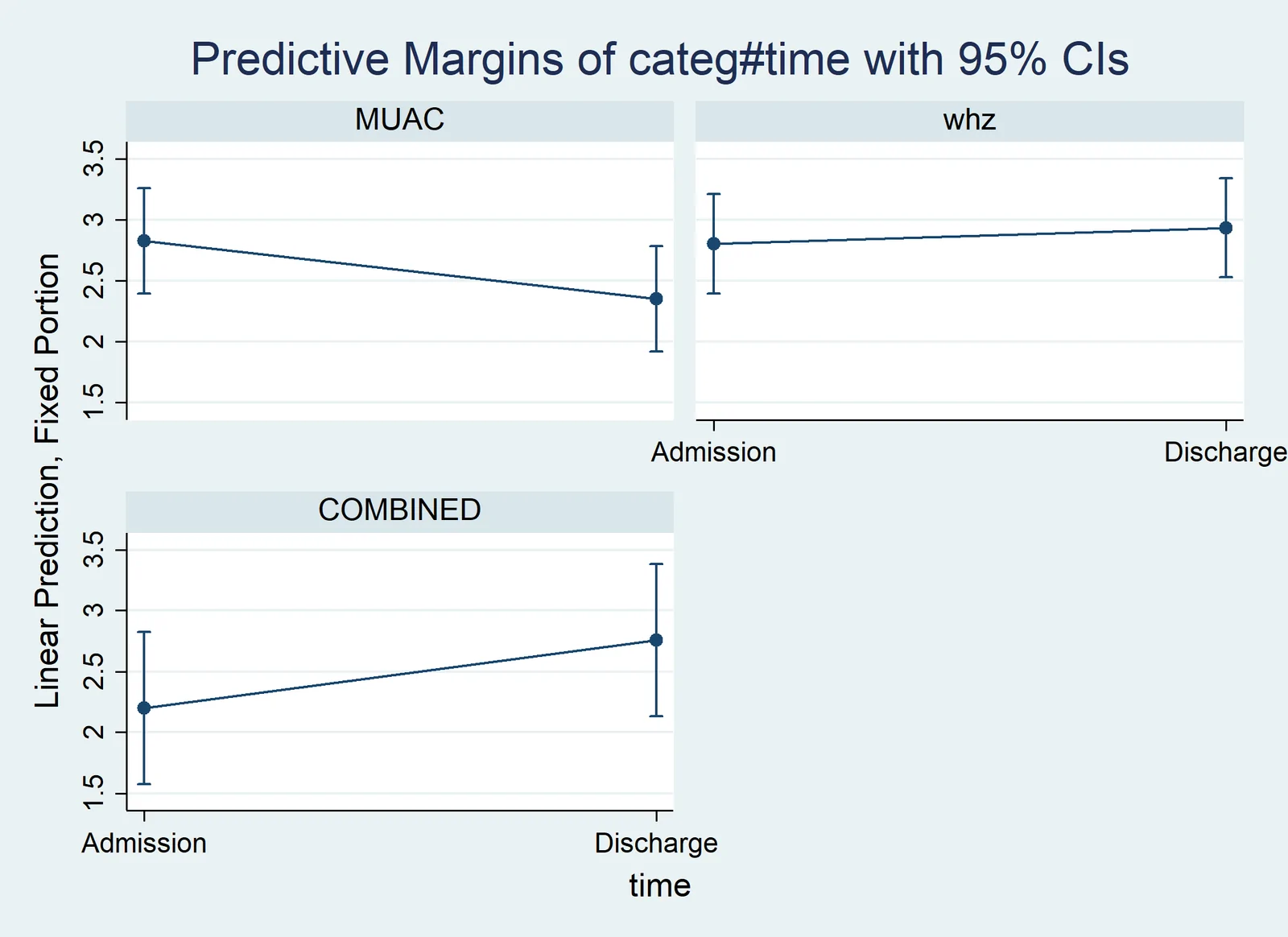
Predicted margins from linear mixed effects models of PhA. Predicted margins of PhA at admission and discharge by admission group. Estimates are from linear mixed effects models accounting for repeated measurements within individuals (n = 127), with predicted values shown with 95% confidence intervals. PhA phase angle; SAM, severe acute malnutrition; OTP, outpatient therapeutic program; MUAC, mid‑upper arm circumference; WHZ, weight‑for‑height z‑score.

TBW percentage values moved in opposite directions across groups: it significantly declined in SAM children of the MUAC group, while it was largely stable/tendency to slightly increase in the other two groups (Table 2, Fig 4). Despite changes in TBW expressed as a percentage of body weight, absolute impedance values remained stable, indicating that shifts reflected changes in body composition rather than hydration instability. Phase angle rose numerically in the children belonging to the Combined group – yet faring at very low absolute levels– and in the WHZ group and decreased pre–post in MUAC (Table 2, Fig 5).

### Between-group differences at discharge

Adjusted analyses at discharge indicated that important differences persisted between admission groups despite all children meeting program recovery criteria. Compared with children admitted by MUAC alone, children admitted by WHZ alone and those meeting both MUAC and WHZ criteria exhibited lower FMI and higher total body water percentage. Children in the Combined group also retained poorer anthropometric status, reflected by lower WAZ values at discharge (Table 7).

**Table 7:** Linear mixed effects models of baseline anthropometric and body‑composition outcomes, with the MUAC group used as reference. Models are restricted to recovered children assessed at discharge (n = 127). Coefficients are shown with 95% confidence intervals and p‑values; models adjusted for age and sex.

	FMI		FFMI		TBW		PhA		WHZ		WAZ		HAZ		TSFZ	
	Coeff (95% CI)	p- value	Coeff (95% CI)	p- value	Coeff (95% CI)	p- value	Coeff (95% CI)	p- value	Coeff (95% CI)	p- value	Coeff (95% CI)	p- value	Coeff (95% CI)	p- value	Coeff (95% CI)	p- value
Intercept	5.200		9.290		50.300		2.340		−1.780		−2.970		−2.930		−1.830	
WHZ Group	−0.943 (−1.462 −0.425)	**<0.001**	0.109 (−0.401 0.618)	0.676	3.900 (0.892 6.908)	**0.011**	0.584 (−0.030 1.197)	0.062	−0.561 (−0.913 −0.211)	**0.002**	0.453 (0.018 0.888)	**0.041**	1.338 (0.686 1.989)	**<0.001**	0.623 (0.198 1.048)	**0.004**
Combined Group	−1.100 (−1.737 −0.462)	**<0.001**	−0.103 (−0.732 0.524)	0.746	5.141 (1.449 8.832)	**0.006**	0.406 (−0.337 1.149)	0.284	−1.017 (−1.446 −0.589)	**<0.001**	−0.572 (−1.094 −0.050)	**0.032**	0.162 (−0.617 0.941)	0.684	0.094 (−0.419 0.608)	0.719
Gender Female	−0.042	0.855	−0.412	0.073	−1.376	0.307	−0.182	0.575	0.157	0.344	0.341	**0.058**	0.617	**0.025**	0.177	0.366
Age	−0.005	0.687	−0.028	**0.018**	−0.071	0.307	0.007	0.484	−0.010	0.107	−0.019	**0.048**	−0.031	**0.033**	0.012	0.186

FMI, fat mass index; FFMI, fat free mass index; TBW, total body water; WHZ, Weight-for-height z-score; WAZ, weight-for-age z-score; HAZ, height-for-age z-score; TSFZ, triceps skinfold thickness z-score.

When the Combined group was used as the reference category, children in both the MUAC and WHZ groups demonstrated more favorable recovery profiles. Children in the MUAC group had higher FMI and better anthropometric outcomes, while children in the WHZ group showed higher WHZ, WAZ, HAZ, and TSFZ values at discharge. Together, these findings suggest that children in the Combined group remained the most nutritionally vulnerable group at recovery, despite achieving standard discharge thresholds (Table 8).

**Table 8:** Linear mixed effects models of baseline anthropometric and body‑composition outcomes, with the Combined group used as reference. Models are restricted to recovered children assessed at discharge (n = 127). Coefficients are shown with 95% confidence intervals and p‑values; models adjusted for age and sex.

	FMI		FFMI		TBW		PhA		WHZ		WAZ		HAZ		TSFZ	
	Coeff (95% CI)	p- value	Coeff (95% CI)	p- value	Coeff (95% CI)	p- value	Coeff (95% CI)	p- value	Coeff (95% CI)	p- value	Coeff (95% CI)	p- value	Coeff (95% CI)	p- value	Coeff (95% CI)	p- value
Intercept	4.070		9.240		55.760		2.750		−2.860		−3.580		−2.620		−1.680	
MUAC Group	1.100 (−0.462 1.737)	**<0.001**	0.103 (−0.524 0.732)	0.746	−5.141 (−9.832 −1.449)	**0.006**	−0.406 (−1.149 0.337)	0.284	1.017 (−0.589 1.446)	**<0.001**	0.572 (-0.500 to 1.094)	**0.032**	−0.162 (−0.941 0.617)	0.684	−0.094 (−0.608 0.419)	0.719
WHZ Group	0.157 (−0.472 0.785)	0.625	0.212 (−0.407 0.831)	0.501	−1.241 (−4.868 2.387)	0.503	0.178 (−0.558 0.913)	0.636	0.455 (−0.324 0.879)	**0.035**	1.025 (−0.509 1.541)	**<0.001**	1.176 (−0.405 1.946)	**0.003**	0.528 (0.021 1.036)	**0.041**
Gender Female	−0.042	0.855	−0.412	0.073	−1.376	0.307	−0.182	0.575	0.157	0.344	0.341	**0.058**	0.617	**0.025**	0.177	0.366
Age	−0.005	0.687	−0.028	**0.018**	−0.071	0.307	0.007	0.484	−0.010	0.107	−0.199	**0.048**	−0.031	**0.033**	0.012	0.186

FMI, fat mass index; FFMI, fat free mass index; MUAC, Mid-upper arm circumference; TBW, total body water; WHZ, Weight-for-height z-score; WAZ, weight-for-age z-score; HAZ, height-for-age z-score; TSFZ, triceps skinfold thickness z-score.

### Differences in changes from admission to discharge (paired-cohort, n=127)

Adjusted analyses of changes during treatment showed distinct recovery trajectories across admission groups. Compared with children admitted by MUAC alone, children admitted by WHZ alone experienced greater improvements in fat-free mass indicators, while showing smaller increases in fat mass. In contrast, changes observed among children in the Combined group were broadly similar to those of the MUAC group, with no statistically significant differences in FMI or FFMI gains (Tables 9 and 10).

**Table 9:** Linear mixed effects models of changes in body‑composition outcomes between admission and discharge among recovered children (n = 127). The MUAC admission group was used as the reference category. Coefficients are presented with 95% confidence intervals and p‑values; models were adjusted for age and sex. These models estimate adjusted differences in admission‑to‑discharge change.

	FMI		FFMI		PhA		TBW	
	Coeff (95% CI)	p- value	Coeff (95% CI)	p- value	Coeff (95% CI)	p- value	Coeff (95% CI)	p- value
WHZ Group	−0.912 (−1.471 −0.354)	**0.001**	0.674 (0.106 1.244)	**0.020**	0.607 (0.052 1.163)	**0.032**	5.094 (1.831 8.355)	**0.002**
Combined Group	−0.515 (−1.262 0.232)	0.177	0.567 (−0.194 1.32)	0.144	1.034 (0.296 1.773)	**0.006**	3.604 (−0.756 7.965)	0.105
Gender Female	0.118	0.538	−0.235	0.362	−0.037	0.880	−0.291	0.794
Age	−0.012	0.888	−0.028	**<0.001**	0.010	0.336	0.070	0.170

FMI, fat mass index; FFMI, fat free mass index; MUAC, Mid-upper arm circumference; TBW, total body water; WHZ, Weight-for-height z-score; PhA, Phase Angle.

**Table 10:** Linear mixed effects models of changes in body‑composition outcomes between admission and discharge among recovered children (n = 127). The Combined admission group was used as the reference category. Coefficients are presented with 95% confidence intervals and p‑values; models were adjusted for age and sex. These models estimate adjusted differences in admission‑to‑discharge change.

	FMI		FFMI		PhA		TBW	
	Coeff	p- value	Coeff	p- value	Coeff	p- value	Coeff	p- value
MUAC Group	0.515 (−0.232 1.262)	0.177	−0.567 (−1.328 0.194)	0.144	−1.034 (−1.773 −0.296)	**0.006**	−3.604 (−7.965 0.756)	0.105
WHZ Group	−0.397 (−1.128 0.334)	0.287	0.108 (−0.637 0.853)	0.776	−0.427 (−1.153 0.298)	0.248	1.489 (−2.771 5.748)	0.493
Gender Female	0.118	0.538	−0.295	0.114	−0.037	0.880	−0.291	0.794
Age	−0.012	0.888	−0.028	**<0.001**	0.010	0.336	0.070	0.170

FMI, fat mass index; FFMI, fat free mass index; MUAC, Mid-upper arm circumference; TBW, total body water; WHZ, Weight-for-height z-score, PhA Phase Angle.

Recovery patterns also differed with respect to phase angle. Relative to the MUAC group, both the WHZ and Combined groups demonstrated greater improvements in phase angle over the course of treatment, suggesting more marked recovery of cellular integrity and tissue function. These findings are consistent with the broader pattern of body-composition recovery observed across the three admission groups.

*Sub-analysis for weight Gain, FMI gain and FFMI gain by age at admission (paired-cohort, n = 127)*

Across three age bands (<12 months, 12–23 months, 24–59 months), FMI gain was largest in infants <12 months (+1.03 ± 1.55 kg/m^2^), smaller in 24–59 months (+0.47 ± 1.23 kg/m^2^), and minimal in 12–23 months (+0.08 ± 1.61 kg/m^2^). When compared with the age category group <12 months, the 12–23 months group had a significantly smaller FMI gain (*p* = 0.002), while there was no significant difference with the age category 24–59 months (*p* = 0.138). Weight gain (kg) was also higher in <12 months (0.80 ± 0.58) than 12–23 months (0.61 ± 0.84; *p* = 0.047), with no difference versus 24–59 months (0.79 ± 0.09; *p* = 0.802). FFMI change did not differ significantly across age categories (all *p* > 0.10) (S2 Table).

## Discussion

This study characterizes specific SAM admission anthropometric groups and body composition on admission and discharge among children meeting SAM admission criteria by MUAC only, WHZ only, or Combined (concurrent presence of MUAC and WHZ admission criteria), and aims at comparing the findings against recent scientific evidence, notably the OptiDiag study and recent trials reporting BIA derived outcomes in children with SAM. On admission, children belonging to the MUAC and Combined groups were younger and more stunted than those in the WHZ group, whereas children of the WHZ group were taller and had larger triceps skinfolds (TSFZ). The children of the Combined group presented the most severe anthropometric deficits (lowest WAZ and WHZ) and the lowest PhA and FMI. These patterns are consistent with the OptiDiag’s multi-country observations where children belonging to the Combined group carried the heaviest burden of functional derangements at admission, with WHZ-only children often older/taller and MUAC-only children younger and more linearly growth restricted.

Growth trajectories during OTP diverged systematically by anthropometric admission group. Children admitted by WHZ criteria only exhibited the fastest recovery (shortest length of stay, highest average weight gain), with predominant FFM accrual (around 82% of weight gain as FFM), a sizable rise in FFMI, and numerically higher PhA at discharge, all suggestive of qualitative tissue restoration beyond that reflected by weight gain. By contrast, children with MUAC only admission criteria accrued a greater proportion of FM (around 78% of gain as FM), very little gain in FFM, and displayed a fall in TBW percentage, while showing no improvement in PhA; this profile suggests preferential fat repletion with limited lean tissue recovery. The children belonging to the Combined group demonstrated intermediate/mixed tissue recovery (limited FM and FFM increases), the longest length of stay, and only modest PhA increase (yet with low absolute values among recovered children), with anthropometric z scores, including WAZ score, remaining most negative at exit.

The children in the MUAC group showed an important and significant decrease in TBW percentage. This is likely the result of a disproportionate fat recovery during treatment (FM moved from 1.81 to 2.38 Kg, and fat percentage increased from 30.1% to 35.3% of the total body weight), which lowered TBW as a percentage of body weight even when absolute value of TBW remained stable (as evidenced by the stable level of the Impedance values between admission and discharge). Physiologically, fat consists of 10–15% water, while lean tissue is around 70–75% of water [14]. As fat in the children in the MUAC group increased faster than lean tissue, the denominator (total body weight) grew, but the water-rich part of the body did not grow as fast during the treatment period, thus reducing TBW percentage values.

Children in the MUAC and Combined groups were younger and more likely to be wasted and stunted, indicating clear evidence of chronic malnutrition. These findings suggest that both children in the MUAC and Combined groups likely experienced a greater degree of fat depletion before admission to the nutrition program, potentially indicating a prolonged and severe catabolic state. The lower TSFZ at admission supports this reasoning. The median duration to progress to SAM is estimated to be around 7.5 months, during which the body starts utilizing mainly fat as the energy source to support life, before turning to protein consumption, which can prelude the development of more extreme catabolic conditions associated with debilitation, starvation, and life-threatening infections [29,30]. It is reasonable, therefore, to have observed and detected in our study a predominant process of fat accumulation in stunted/younger groups of infants/children with SAM during the treatment period, as this represents the normal physiological rehabilitation process for this group of children. In contrast, the children in the WHZ/wasted/older group, growing and more active (walking, running, climbing) during the treatment period, were likely to burn fat mass and develop greater muscle mass because of their developmental stage.

These specific characteristics of the anthropometric admission groups align closely with prior body composition studies in outpatient SAM. Kangas et al. [16], who studied children with SAM during their recovery trajectory in a nutrition program testing different types of RUTF (without differentiating the children in different groups according to the MUAC and WHZ criteria, as done in this study), reported that roughly half of weight gain was FFM across RUTF dosing arms, with incomplete FM catch up at program recovery. This outcome reflects an ‘efficiency’ pattern most closely resembling the lean directed gains in the WHZ only cohort of the present study.

At discharge, all groups showed improvements in weight and MUAC. However, the relative differences across the three different groups persisted: differences in WHZ values continued to reflect those seen during admission, with the MUAC‑admitted group having the highest WHZ values, followed by the WHZ group, and then the Combined group. Linear growth also remained poor across all groups, with HAZ ≤ −2 overall, although this was most pronounced among children in the MUAC and Combined groups, underscoring persistent linear growth failure. Severe underweight (WAZ < −3 Z score) persisted in the children of the Combined group.

Phase angle (PhA) offered complementary and integrative inferential insights on functional recovery. In our cohort, baseline PhA was lowest in the children of the Combined group, followed by the children in the MUAC group; with treatment, PhA improved both in the WHZ group and Combined group, and did not improve in children belonging to the MUAC group. Systematic reviews in pediatrics have shown that PhA robustly correlates with nutritional status, muscle mass, fat free mass, and morbidity risk across diverse pediatric illnesses, including malnutrition. Among hospitalized or clinically vulnerable children, low PhA is consistently associated with greater complication rates, prolonged hospital stays, and higher mortality risk. In malnutrition specifically, Girma and colleagues [24] demonstrated that PhA and impedance vector position improve during SAM treatment and sensitively reflect shifts in hydration and cellular recovery, even in edematous children, where anthropometry loses reliability.

By discharge, the differences described in our study culminated in divergent recovery profiles. Children belonging to the WHZ group appeared to reach a comparatively robust state, with more favorable FFM, FMI, and PhA patterns than children of the MUAC group. This most likely reflected the capacity of older, less stunted children to rebuild tissue more efficiently. Children of the MUAC group in contrast, reached anthropometric discharge criteria but retained signs of poor FFMI and PhA pattern recovery. Children in the Combined group showed a weak trajectory recovery of the BIA parameters that, associated with the persistence of severe WAZ score from admission to discharge, confirmed the absolute vulnerability of this group. In a recent analysis of cohort data from Senegal, a combination of a severely low Mid-upper arm circumference (MUAC < 11.5 cm) and a severely low WAZ (i.e., WAZ <−2.8) detected all near-term (i.e., occurring within 6 months of measurement) deaths associated with either a weight-for-height z-score (WHZ) <−3 and/or concurrent wasting and stunting (WHZ <−2 and height-for-age z-score (HAZ) <−2) [31,32].

A key strength of this study is its novel, direct comparison of children with SAM admitted by MUAC only, WHZ only, or combined MUAC+WHZ criteria within the same programmatic setting. By disaggregating children with SAM into these three admission groups, and by integrating BIA-derived indicators, the study enables a complementary assessment of recovery dynamics beyond conventional anthropometry alone.

Data were collected in a real‑world outpatient therapeutic program (OTP) operating under routine CMAM guidelines in a high‑burden, resource‑limited context, enhancing external validity and programmatic relevance.

This study has several important limitations. First, because longitudinal analyses were restricted to children discharged as recovered, the findings reflect body-composition trajectories among treatment completers rather than all children admitted with SAM. Children who defaulted, failed treatment, or required referral may have experienced different recovery patterns. Consequently, survivor bias cannot be excluded and the results should not be generalized to the entire population of children admitted with severe acute malnutrition.

Second, the study is observational, and comparisons between groups (MUAC, WHZ, and Combined) are subject to residual confounding. The groups differed at baseline in key characteristics, including age, sex distribution, and anthropometric severity. Although analyses were adjusted for age and sex, residual confounding remains possible. Data on household food security, socioeconomic status, infant and young child feeding practices, prior episodes of acute malnutrition, and the burden of subclinical infection were not available. These factors may influence both body composition at admission and recovery trajectories during treatment. Consequently, differences observed between admission groups may partly reflect underlying social, environmental, and clinical factors rather than differences attributable solely to anthropometric admission criteria.

A third limitation concerns the Combined group, for which discharge decisions followed routine programmatic practice and could be based on either MUAC or WHZ recovery criteria. Consequently, children within this group may not have reached identical anthropometric recovery thresholds at discharge, potentially increasing within-group heterogeneity and affecting comparisons with the MUAC-only and WHZ-only groups.

Fourth, the use of bioelectrical impedance analysis (BIA) introduces measurement‑related limitations. While BIA provides a feasible, non‑invasive method for assessing body composition in programmatic settings, its estimates in young children with SAM may be influenced by hydration status, tissue composition, and developmental stage. Although children with edema were excluded to minimize distortion, BIA‑derived measures (e.g., FM, FFM, and PhA) remain indirect estimates. Moreover, PhA showed substantial inter‑individual variability, particularly across age groups, contributing to relatively large standard deviations despite stable group means. Overall, the study lacked a gold‑standard body composition reference method, such as dual‑energy X‑ray absorptiometry (DEXA) or isotope dilution, which limits the ability to formally validate BIA‑derived estimates [14]. Consequently, absolute body‑composition values should be interpreted with caution: emphasis should be placed on within‑child changes and between‑group comparisons rather than precise quantification, while interpretations related to lean tissue restoration and body-composition recovery patterns should be considered interpretative inferences rather than definitive biological outcomes. Overall, the BIA-derived parameters reported here should be interpreted as exploratory markers of body composition rather than definitive measures of physiological recovery.

Finally, the study was unable to assess long‑term outcomes following program discharge, including relapse, morbidity, mortality, and sustained tissue recovery, due to premature termination of follow‑up during the COVID‑19 pandemic. As a result, inferences regarding post‑discharge vulnerability remain indirect and hypothesis‑generating.

## Conclusion

In this prospective cohort, children admitted to outpatient treatment for SAM by MUAC only, WHZ only, or combined MUAC and WHZ criteria exhibited distinct patterns of tissue accretion and recovery during treatment, despite all meeting current anthropometric discharge criteria. Although causal inference is precluded by the observational design, the internal consistency of anthropometric, body‑composition, and bio‑impedance indicators suggests that recovery following SAM treatment is not uniform across admission groups.

Importantly, these contrasting recovery profiles were closely linked to underlying differences in age and chronic undernutrition across admission groups: children admitted by WHZ criteria tended to demonstrate recovery profiles characterized by predominant fat‑free mass accretion, larger gains in FFMI, and numerically higher phase angle values at discharge, consistent with a pattern of comparatively efficient lean tissue restoration. In contrast, children admitted by MUAC alone accumulated proportionally more fat mass, showed smaller gains in lean tissue, and experienced little or no improvement in phase angle, despite achieving anthropometric recovery. Children meeting both MUAC and WHZ criteria showed the slowest and most constrained recovery trajectories, with persistent underweight and low bio‑impedance values at discharge.

These findings should be considered exploratory and hypothesis-generating because relapse, mortality, functional outcomes, and post-discharge body-composition trajectories were not assessed. Consequently, the results do not support modifications to existing discharge criteria. Rather, they highlight the need for longitudinal studies evaluating whether body-composition differences at discharge predict subsequent clinical outcomes and post-discharge vulnerability.

## Supporting information

S1 Fig Predicted margins from linear mixed effects models of FMI. Predicted margins of fat mass index (FMI) at admission and discharge by SAM admission group (MUAC, WHZ, and Combined), with 95% confidence intervals.
(DOCX)

S2 Fig Predicted margins from linear mixed effects models of FFMI. Predicted margins of fat-free mass index (FFMI) at admission and discharge by SAM admission group (MUAC, WHZ, and Combined), with 95% confidence intervals.
(DOCX)

S1 Table Baseline anthropometric and body-composition characteristics of children with severe acute malnutrition at admission. Descriptive comparison of demographic, anthropometric, bioelectrical impedance, and body-composition indicators across MUAC-only, WHZ-only, and Combined admission groups.
(DOCX)

S2 Table Age-stratified analysis of weight gain, fat mass index gain, and fat-free mass index gain during recovery. Comparison of recovery patterns by age category among recovered children with severe acute malnutrition.
(DOCX)

S1 File Anonymized study dataset. (XLSX)

## Abbreviations

CMAM: community-based management of acute malnutrition
FFM: fat-free mass
FFMI: fat-free mass index
FM: fat mass
FMI: fat mass index
HAZ: height-for-age z-score
MAM: moderate acute malnutrition
MUAC: mid-upper arm circumference
OTP: outpatient therapeutic program
PhA: phase angle
RUSF: ready-to-use supplementary food
RUTF: ready-to-use therapeutic food
SAM: severe acute malnutrition
SC: Stabilization Center
TSF: triceps skinfold thickness
TSFP: targeted supplementary feeding program
UNICEF: United Nation Children’s Fund
WAZ: weight-for-age z-score
WHO: World Health Organization
WHZ: weight-for-height z-score
