# Supplementary material for: Association between anthropometric criteria and body composition among children aged 6–59 months with uncomplicated severe acute malnutrition: A prospective cohort study from South Sudan
Source: PLoS One. 2026 Sep 15;21(9):e0356530. doi: 10.1371/journal.pone.0356530 (PMC13577578; doi:10.1371/journal.pone.0356530)
Supplement: Supplementary file 1 [file pone.0356530.s001.docx]

**S1 Fig. Change in fat mass index (DeltaFMI) from admission to discharge in the paired cohort of children (n=127) with severe acute malnutrition (SAM).**

The figure presents the mean change in FMI between admission and discharge among the 127 children with SAM who recovered following outpatient therapeutic program (OTP) treatment and had paired measurements at both time points.

DeltaFMI, change in Fat Mass Index; SAM, severe acute malnutrition; OTP, outpatient therapeutic program; MUAC, mid‑upper arm circumference; WHZ, weight‑for‑height z‑score.
