# Supplementary material for: Association between anthropometric criteria and body composition among children aged 6–59 months with uncomplicated severe acute malnutrition: A prospective cohort study from South Sudan
Source: PLoS One. 2026 Sep 15;21(9):e0356530. doi: 10.1371/journal.pone.0356530 (PMC13577578; doi:10.1371/journal.pone.0356530)
Supplement: Supplementary file 3 [file pone.0356530.s003.docx]

**S1 Table. Descriptive demographic, anthropometric, and body‑composition characteristics of children with severe acute malnutrition (SAM) at admission (n = 194) and at discharge among those classified as recovered (n = 127).**

Data are presented for all children assessed at admission (n = 194) and for those who met recovery criteria and were discharged from the OTP (n = 127). Participants are stratified according to the admission criteria used for SAM diagnosis: mid‑upper arm circumference (MUAC) only, weight‑for‑height z‑score (WHZ) only, or combined MUAC and WHZ criteria. All values are reported as mean ± SD; 95% confidence intervals are shown in parentheses where indicated. Length of stay (LoS) and average weight gain (AWG) are reported for recovered children only. This table presents descriptive summaries only and does not reflect paired or adjusted analyses.

|  |  | MUAC  Group | Combined Group | WHZ  Group |
| --- | --- | --- | --- | --- |
| **Total Admissions** | **194** | **67** | **51** | **76** |
| **Total Recovered** | **127** | **49** | **21** | **57** |
|  |  |  |  |  |
| **Sex, female (N and %)** | Admission | 42 (62.7%) | 23 (45.1%) | 18 (23.7%) |
|  | Discharge | 32 (65.3%) | 9 (42.9%) | 13 (22.8%) |
|  |  |  |  |  |
| **Age in months,**  **Mean ± SD (95% CI), p-value** | Admission | 11.82 **±** 8.09 (9.87 – 13.77) | 15.41 **±** 11.19 (12.32 – 18.50) | 19.70 **±** 10.27 (17.37 – 22.02) |
|  | Discharge | 14.24 **±** 9.42 (11.58 -16.92) | 16.38 **±** 10.60 (11.80 – 20.96) | 21.98 **±** 11.16 (19.05 – 24.91) |
|  | p-value | 0.140 | 0.735 | 0.223 |
|  |  |  |  |  |
| **Age in months (Range), Mode** | Admission | (6-36), 6 | (6-48), 6 | (6-54), 24 |
|  | Discharge | (7-44), 7 | (7-50), 8 | (7-55),15 |
|  |  |  |  |  |
| **Height, cm,**  **Mean± SD (95% CI), p-value** | Admission | 67.1 **±** 6.3 (65.56 – 68.60) | 69.8 **±** 9.60 (67.18 – 72.47) | 76.9 **±** 8.4  (74.99 – 78.79) |
|  | Discharge | 68.1 **±** 6.1 (66.35 – 69.81) | 70.8 **±** 8.6  (67.08 – 74.53) | 78.1 **±** 8.3 (75.87 – 80.23) |
|  | p-value | 0.394 | 0.686 | 0.428 |
|  |  |  |  |  |
| **Weight, Kg,**  **Mean ± SD (95% CI)** **), p-value** | Admission | 6.08 **±** 1.06 (5.82 – 6.33) | 5.95 **±** 1.61 (5.50 – 6.40) | 7.49 **±** 1.44 (7.16 – 7.81) |
|  | Discharge | 6.74 **±** 1.14 (6.42 – 7.06) | 6.73 **±** 1.64 (6.02 – 7.44) | 8.29 **±** 1.44 (7.91 – 8.66) |
|  | p-value | **0.002** | 0.067 | **0.002** |
|  |  |  |  |  |
| **MUAC, cm,**  **Mean ± SD (95% CI), p-value** | Admission | 11.08 **±** 0.36 (10.99 -11.16) | 10.76 **±** 0.68 (10.57 – 10.95) | 12.02 **±** 0.64 (11.88 – 12.17) |
|  | Discharge | 11.70**±** 0.51 (11.56 – 11.85) | 11.67 **±** 0.38 (11.51 – 11.84) | 12.32 **±** 0.55 (12.17 – 12.46) |
|  | p-value | **<0.001** | **<0.001** | **0.006** |
|  |  |  |  |  |
| **TSF, mm,**  **Mean ± SD (95% CI)** **), p-value** | Admission | 5.05 **±** 0.86 (4.84 – 5.26) | 4.55 **±** 0.85 (4.31 – 4.80) | 5.89 **±** 1.28 (5.60 – 6.18) |
|  | Discharge | 5.80 **±** 1.18 (5.47 - 6.14) | 5.73 **±** 0.79 (5.39 – 6.08) | 6.28 **±** 1.20 (5.97 – 6.60) |
|  | p-value | **<0.001** | **<0.001** | 0.077 |
|  |  |  |  |  |
| **HAZ,**  **Mean ± SD (95% CI)** **), p-value** | Admission | -2.20 **±**0.75 (-2.63 -1.78) | -2.54 **±** 1.52  (-2.97 -2.10) | -1.77 **±** 1.56  (-2.12 -1.41) |
|  | Discharge | -2.74 **±** 1.66 (-3.21 -2.27) | -2.84 **±** 1.19 (-3.36 -2.33) | -2.02**±** 1.59 (-2.43 -1.61) |
|  | p-value | 0.098 | 0.426 | 0.364 |
|  |  |  |  |  |
| **WAZ,**  **Mean ± SD ((95% CI)** **), p-value** | Admission | -3.30 **±** 1.11 (-3.77 -3.03) | -4.45 **±** 1.19  (-4.77 -4.12) | -3.40 **±** 1.01  (-3.65 -3.15) |
|  | Discharge | -2.91**±** 1.17 (-3.24 -2.58) | -3.64**±** 0.87 (-4.01 -3.25) | -2.81**±** 0.97  (-3.07 -2.56) |
|  | p-value | 0.072 | **0.006** | **0.001** |
|  |  |  |  |  |
| **WHZ,**  **Mean ± SD (95% CI), p-value** | Admission | -2.66 **±** 1.11 (-2.93 -2.40) | -4.10 **±** 0.83 (-4.33 -3.87) | -3.49 **±** 0.69  (-3.64 -3.33) |
|  | Discharge | -1.71 **±** 1.00 (-1.99 -1.43) | -2.81 **±** 0.72 (-3.12 -2.50) | -2.45 ±0.68 (-2.63 -2.28) |
|  | p-value | **<0.001** | **<0.001** | **<0.001** |
|  |  |  |  |  |
| **TSFZ,**  **Mean ± SD (95% CI), p-value** | Admission | -2.78 **±** 0.88) (-2.99 -2.56) | -3.31 **±** 1.24)  (-3.67 -2.96) | -1.69 **±** 1.06 (-1.95 -1.42) |
|  | Discharge | -1.93**±** 1.01) (-2.22 -1.64) | -1.85 **±** 0.78) (-2.19 -1.51) | -1.29 **±** 1.03 (-1.56 -1.01) |
|  | p-value | **<0.001** | **<0.001** | **0.041** |
|  |  |  |  |  |
| **Length of Stay (LoS), days** |  | 46.5 | 51.9 | 30.6 |
|  |  |  |  |  |
| **Average Weight Gain (AWG) g/Kg/day** |  | 2.62 | 2.81 | 3.06 |
|  |  |  |  |  |
| **FFM, kg,**  **Mean ± SD (95% CI), p-value** | Admission | 4.19 **±** 0.87 (3.98 – 4.40) | 4.26 **±** 1.24  (3.92 -4.61) | 5.08 **±** 1.13  4.82 – 5.33) |
|  | Discharge | 4.36 **±** 0.82 (4.13 – 4.59) | 4.66 **±** 1.07 (4.20 – 5.12) | 5.74 **±** 1.29 (5.40 – 6.07) |
|  | p-value | 0.301 | 0.208 | **0.002** |
|  |  |  |  |  |
| **FM, Kg,**  **Mean ± SD (95% CI), p-value** | Admission | 1.87 **±** 0.76 (1.69 -2.05) | 1.75 **±** 0.77 (1.53 – 1.96) | 2.41 **±** 0.82  (2.22 – 2.60) |
|  | Discharge | 2.38 **±** 0.65 (2.20 – 2.56) | 2.07**±** 0.82 (1.72 – 2.43) | 2.55 **±** 0.91 (2.31 – 2.79) |
|  | p-value | **<0.001** | 0.112 | 0.351 |
|  |  |  |  |  |
| **Fat % [FM/Weight *100]** | Admission | 30.8% | 29.4% | 32.2% |
|  | Discharge | 35.3% | 30.8% | 30.8% |
|  |  |  |  |  |
| **FFMI, Kg/m^2,^**  **Mean ± SD (95% CI), p-value** | Admission | 9.30 **±** 1.43 (8.96 – 9.65) | 8.64 **±** 1.04 (8.35 – 8.92) | 8.56 **±** 1.18 (8.29 – 8.83) |
|  | Discharge | 9.38 **±** 1.12 | 9.26**±** 0.23 | 9.38 **±** 1.43 |
|  | p-value | 0.753 | **0.022** | **<0.001** |
|  |  |  |  |  |
| **FMI, Kg/m^2^**  **Mean ± SD (95% CI), p-value** | Admission | 4.13 **±** 1.47 (3.77 – 4.48) | 3.57 **±** 1.63 (3.12 – 4.02) | 4.03 **±** 1.18 (3.79 – 4.30) |
|  | Discharge | 5.11 **±** 1.03 (4.82 – 5.41) | 4.04 **±** 0.97 (3.62 – 4.46) | 4.21**±** 1.46 (3.83 – 4.59) |
|  | p-value | **<0.001** | 0.223 | 0.458 |
|  |  |  |  |  |
| **TBW %,**  **Mean ± SD (95% CI), p-value** | Admission | 54.51 **±** 7.98 (52.60 – 56.44) | 56.28 **±** 6.35 (54.53 – 58.01) | 53.25**±** 6.75 (51.73 – 54.77) |
|  | Discharge | 50.82**±** 5.39 (49.29 - 52.37) | 55.85**±** 9.09 (51.93 – 59.78) | 54.28**±** 7.63  (52.28 – 56.28) |
|  | p-value | **0.006** | 0.818 | 0.413 |
|  |  |  |  |  |
| **Impedance Ω,**  **Mean ± SD (95% CI), p-value** | Admission | 945.46 **±** 143.41 (919.91 – 989.02) | 1009.20 **±** 112.97 (978.00 – 1040.40) | 1041.21**±** 134.47 (1010.79 – 1071.63) |
|  | Discharge | 946.06 **±** 136.38 (907.51 -984.62) | 942.38 **±** 138.59 (882.53 – 1002.23) | 939.96 **±** 139.31 (903.45 -976.48) |
|  | p-value | 0.751 | **0.036** | **<0.001** |
|  |  |  |  |  |
| **PhA,**  **Mean ± SD (95% CI), p-value** | Admission | 2.9 **±** 1.4 (2.6 – 3.2) | 2.2 **±** 0.8 (2.0 – 2.4) | 2.8 **±** 1.6 (2.5 – 3.2) |
|  | Discharge | 2.3 **±** 0.8 (2.1 – 2.5) | 2.7**±** 1.5 (2.1 -3.4) | 3.0**±** 2.0 (2.5 – 3.5) |
|  | p-value | **0.008** | **0.044** | 0.631 |

SAM, severe acute malnutrition; OTP, outpatient therapeutic program; SD, standard deviation; CI, Confidence interval; MUAC, Mid-upper arm circumference; WHZ, Weight-for-height z score; TSF, triceps skinfold thickness; TSFZ, triceps skinfold thickness z score; HAZ, Height-for-age z score; WAZ, Weight-for-age z score; TBW, total body water; FM, fat mass; FMI, fat mass index; FFM, fat free mass; FFMI, fat free mass index; PhA, phase angle.
