# Supplementary material for: Association between anthropometric criteria and body composition among children aged 6–59 months with uncomplicated severe acute malnutrition: A prospective cohort study from South Sudan
Source: PLoS One. 2026 Sep 15;21(9):e0356530. doi: 10.1371/journal.pone.0356530 (PMC13577578; doi:10.1371/journal.pone.0356530)
Supplement: Supplementary file 4 [file pone.0356530.s004.docx]

**S2 Table. Linear mixed effects models to assess age‑group differences in changes from admission to discharge among children (n=127) with severe acute malnutrition (SAM).**

Values are presented as mean change (SD) from admission to recovery, stratified by age group (<12 months, 12–23 months, and 24–59 months). Linear mixed effects models were used to assess differences in changes across age groups, with children aged <12 months as the reference category. Models included a random intercept for each child to account for within‑subject correlation of repeated measurements (admission and recovery) and were adjusted for age and sex. Regression coefficients represent the mean difference in change compared with the reference group, with 95% confidence intervals (CI) and corresponding p‑values reported.

| ***Linear Mixed-Effects Models***  ***(reference value Cat <12 months)*** |  | **<12 months**  **N=55** | **12-23 months**  **N=36** | **24-59 months**  **N=36** | ***Groups comparison*** | | ***Coefficient***  ***(95% CI)*** | ***p-value*** |
| --- | --- | --- | --- | --- | --- | --- | --- | --- |
| **FMI Change Kg/m^2^, Mean (SD)** | From Admission to Recovery | 1.03 (1.55) | 0.08 (1.61) | 0.47 (1.23) | 12-23m  24-59m | -0.93 (-1.54 -0.33)  -0.46 (-1.06 0.15) | | **0.002**  0.138 |
|  |  |  |  |  |  |  | |  |
| **FFMI Change Kg/m^2^, Mean (SD)** | From Admission to Recovery | 9.26 (1.10) | 8.89 (1.41) | 8.45 (1.33) | 12-23m  24-59m | -0-44 (-1.15 1.03)  -0.40 (-0.19 0.98) | | 0.142  0.185 |
|  |  |  |  |  |  |  | |  |
| **Weight Gain, kg, Mean (SD)** | From admission to Recovery | 0.80 (0.58) | 0.61 (0.84) | 0.79 (0.09) | 12-23m  24-59m | -0.21 (-0.41 -0.01)  0.01 (-0.19 0.21) | | **0.047**  0.931 |
|  |  |  |  |  |  |  | |  |

SAM, severe acute malnutrition; OTP, outpatient therapeutic program; SD, standard deviation; CI, Confidence Interval; FMI, fat mass index; FFMI, fat‑free mass index.
